# The Dynamic Mortise-and-Tenon Interlock Assists Hydrated Soft Robots Toward Off-Road Locomotion

**DOI:** 10.34133/research.0015

**Published:** 2022-12-19

**Authors:** Baoyi Wu, Yaoting Xue, Israt Ali, Huanhuan Lu, Yuming Yang, Xuxu Yang, Wei Lu, Yinfei Zheng, Tao Chen

**Affiliations:** ^1^ Key Laboratory of Marine Materials and Related Technologies, Zhejiang Key Laboratory of Marine Materials and Protective Technologies, Ningbo Institute of Material Technology and Engineering, Chinese Academy of Sciences, Ningbo 315201, China.; ^2^ School of Chemical Sciences, University of Chinese Academy of Sciences, 19A Yuquan Road, Beijing 100049, China.; ^3^ Department of Engineering Mechanics, Zhejiang University, Hangzhou 310027, China.; ^4^ INRS-EMT, 1650 Boul. Lionel Boulet, Varennes J3X 0A1, Canada.; ^5^ College of Chemical Engineering, Ningbo Polytechnic, Ningbo 315800, China.; ^6^ Key Laboratory for Biomedical Engineering of Ministry of Education Ministry of China, Key Laboratory of Clinical Evaluation Technology for Medical Device of Zhejiang Province, College of Biomedical Engineering and Instrument Science, Zhejiang University, Hangzhou 310027, China.; ^7^ Research Center for Humanoid Sensing, Zhejiang Lab, Hangzhou 311100, China.

## Abstract

Natural locomotion such as walking, crawling, and swimming relies on spatially controlled deformation of soft tissues, which could allow efficient interaction with the external environment. As one of the ideal candidates for biomimetic materials, hydrogels can exhibit versatile bionic morphings. However, it remains an enormous challenge to transfer these in situ deformations to locomotion, particularly above complex terrains. Herein, inspired by the crawling mode of inchworms, an isotropic hydrogel with thermoresponsiveness could evolve to an anisotropic hydrogel actuator via interfacial diffusion polymerization, further evolving to multisection structure and exhibiting adaptive deformation with diverse degrees of freedom. Therefore, a dynamic mortise-and-tenon interlock could be generated through the interaction between the self-deformation of the hydrogel actuator and rough terrains, inducing continual multidimensional locomotion on various artificial rough substrates and natural sandy terrain. Interestingly, benefiting from the powerful mechanical energy transfer capability, the crawlable hydrogel actuators could also be utilized as hydrogel motors to activate static cargos to overstep complex terrains, which exhibit the potential application of a biomimetic mechanical discoloration device. Therefore, we believe that this design principle and control strategy may be of potential interest to the field of deformable materials, soft robots, and biomimetic devices.

## Introduction

Hydrogel actuators, one type of emerging hydrated soft robots, are capable of responding to external stimuli accompanied by producing the reversible morphological change with the transformation from internal chemical energy to external mechanical energy [1–4]. Because of the self-deformation and soft-tissue-like mechanical property, such materials could imitate biological behavior and have been applied in the field, ranging from biomimetic devices to hydrated soft robots [5–12]. Although the existing hydrogel actuators have been able to generate diverse biomimetic morphologies [13–17], it remains a grand scientific challenge to activate this in situ deformation to autonomous locomotion and actuate multiple tasks like other types of soft robots.

In contrast, living organisms have evolved to various types of locomotion such as walking, crawling, and swimming to adapt to the change in the external environment within thousands of years of evolution. For instance, an inchworm could use simple body bending to crawl above rough substrate via alternating friction [18]. In detail, the anterior section of the inchworm would first hold on to the substrate. Then, the body of the inchworm would bend, which would induce the posterior section forward. Subsequently, the posterior section would hold on to the substrate while the anterior section was released, generating anisotropic friction. After the body recovers to the initial shape, the anterior section moves forward and activates the next cycle.

These fantastic phenomena have motivated the structural design of hydrated soft robots that could realize locomotion via the interaction between self-deformation and the external environment [19–22]. For example, when a bilayer hydrogel actuator was placed on a customized ratchet floor, the anterior section of the bilayer hydrogel would be anchored to the ratchet floor via a mortise-and-tenon interlock. In contrast, the posterior section of the bilayer hydrogel does not interact with the ratchet floor, inducing an asymmetrical force and activating the unidirectional motion of the whole hydrogel [23–25]. Recently, Li et al. [26] built millimeter-scale ratcheted structures at the bottom of the anterior section of the bilayer hydrogel actuator. Thus, this hydrogel actuator exhibited potential unidirectional locomotion above rough substrates by the exact mechanism.

Compared with living organisms that could adapt to complex terrains and freely move in the 2-dimensional (2D) or 3-dimensional (3D) space, the existing self-deformation soft robots still lack such motion and could only perform 1-dimensional (1D) unidirectional locomotion [27–32]. In addition, once these samples have been prepared, they could only be appropriate for a particular substrate because of the static and unreconfigurable locomotion mode [33,34]. Therefore, there is still a considerable gap between the existing soft robots and living organisms regarding all-terrain off-road locomotion.

**Fig. 1.**
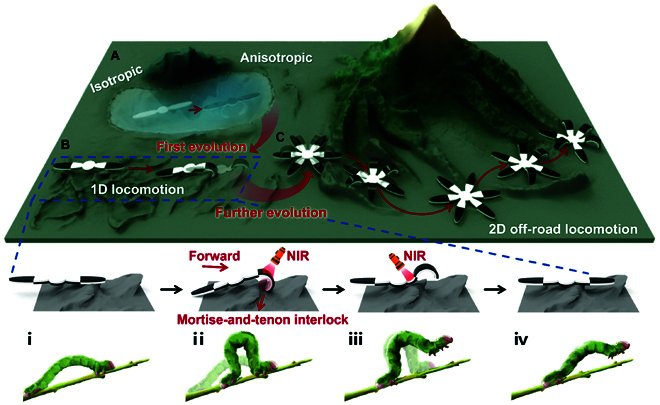
The adaptive evolution and all-terrain locomotion of the PNIPAm sponge. (A) An isotropic PNIPAm sponge could be endowed with an anisotropic structure by growing a passive hydrogel layer via IDP after preparation. (B) The bilayer hydrogel was cut into 3 blocks and reassembled via IDP to form the new anisotropic structure. Based on the anisotropic structure, the linear hydrogel actuator was capable of moving on a rugged substrate via adaptive deformation like the inchworm crawling above branches. (C) The linear hydrogel actuator would evolve into the hexapod actuator and pass through the complex and narrow space.

Herein, we developed a universal strategy—interfacial diffusion polymerization (IDP)—that could reconfigure the anisotropic structure of as-prepared hydrogels, further evolving to multidimensional off-road locomotion. Unlike traditional technologies where the anisotropic structures of the hydrogel actuator are usually designed and fabricated within the preparation process, our designed isotropic poly(*N*-isopropyl acrylamide) (PNIPAm) sponge could directly grow a new photothermal hydrogel layer containing Fe_3_O_4_ nanoparticles to form an anisotropic structure. Furthermore, after preparation, it could get rid of isotropic deformation mode (Fig. 1A). Besides, using the IDP approach, the anisotropic structure of the bilayer hydrogel could be reconfigured, thus obtaining higher degrees of freedom to adapt to the change in external requirements.

With the assistance of programmed near-infrared (NIR) light, the anterior section of the reconfigured hydrogel actuator could firmly anchor into the rough substrate via self-deformation, which induced anisotropic friction and activated forward locomotion. Subsequently, the mortise-and-tenon interlock could be released when the NIR is irradiated onto the mid-section. After removing NIR, the deformed hydrogel actuator could recover to its initial condition and begin the next locomotion cycle (Fig. 1B). In addition, after evolving more tentacles, it could further exhibit adaptive 2D locomotion and pass through complex and narrow terrains such as mountains, ridges, and valleys with the synergy of every tentacle (Fig. 1C). Interestingly, benefiting from the powerful thermoresponsive behavior of the PNIPAm sponge, the hydrogel actuator could be applied as a motor to actuate a static cargo. Using this strategy, a mechanical discoloration device could be fabricated and realize lossless mechanical discoloration. We believe that this work may motivate the development and fabrication of next-generation soft robots with adaptive shape-changing properties and expand more corresponding applications.

## Results

### Thermoresponsive PNIPAm sponge

In order to prepare a powerful stimuli-responsive hydrate material, a PNIPAm sponge with fast thermoresponsiveness was fabricated via the ice-template method [35]. In brief, the hydrogel precursor containing *N*-isopropyl acrylamide (NIPAm) as monomer, ammonium persulfate (APS) as initiator, *N*,*N*,*N*',*N*'-tetramethylethylenediamine (TEMED) as accelerator, and *N*,*N*′-methylenebis(acrylamide) (BIS) as cross-linker was placed at −30 °C in order to generate ice crystals. Subsequently, the hydrogel was polymerized at −5 °C. Thus, a PNIPAm sponge was obtained after removing the ice template at room temperature (Fig. S1 and Movie S1). Compared to the reported ordinary PNIPAm hydrogels, the PNIPAm sponge exhibited excellent thermoresponsiveness in the actuating velocity and deformation scale due to the higher concentration of free water (Fig. S2). As shown in Fig. S3, the dehydrated PNIPAm sponge and ordinary PNIPAm hydrogel were placed in the petri dish containing red dye. Benefiting from the microscope and an open pore structure, the PNIPAm sponge was capable of quickly absorbing water from the petri dish and thus increasing its volume. By contrast, it is difficult for PNIPAm hydrogels to absorb water due to the small and closed pore structure. Similarly, when the PNIPAm sponge and ordinary PNIPAm hydrogel were immersed in red dye, the red dye could be easily washed repeatedly by squeezing the PNIPAm sponge. In contrast, the ordinary PNIPAm hydrogel cannot be squeezed, which indicates that most of the water in the PNIPAm sponge was free water and thus could be quickly transported between the hydrogel and the external environment via the volume phase transition of PNIPAm (Fig. S4).

In practice, PNIPAm hydrogels could transfer the internal chemical energy to mechanical energy via reversible thermoresponsive deformation. Thus, it has usually been utilized as a hydrogel muscle to replicate the biomimetic functions of muscle [36–38]. A PNIPAm sponge was connected to a standard weight without any tension to measure the exported energy quantitatively. According to Newton's laws of motion, the supporting force of electronic balance, *F*_N1_, was equal to the gravity of weight, *G*. When the PNIPAm sponge was shrunk, a tension, *T*, was generated. In this situation, according to the formula *G* = *T* + *F*_N2_, the value of the tension generated within the deformation process could be measured and defined as thermo-induced output force. As shown in Fig. S5, with the thickness increasing, the exported force of the PNIPAm sponge would increase from 24 to 84 mN. Compared with the reported ordinary PNIPAm hydrogel that usually exported 20 mN force with 1 mm thickness, the PNIPAm sponge could export more mechanical force (46 mN) in the same thickness. Moreover, to further evaluate the tension source during the deformation process, a shrinking PNIPAm sponge was connected to the same weight and extruded to the original length by an external force defined as tensile-induced output force. As a result, the value of the thermo-induced output and the tensile-induced output was almost equal, indicating that the shrinkage stress was sourced from stretching the PNIPAm chain. Therefore, the PNIPAm sponge with a larger deformation (deswelling about 40%) could generate more mechanical energy than the ordinary hydrogel (deswelling about 80%) in the same thickness.

**Fig. 2.**
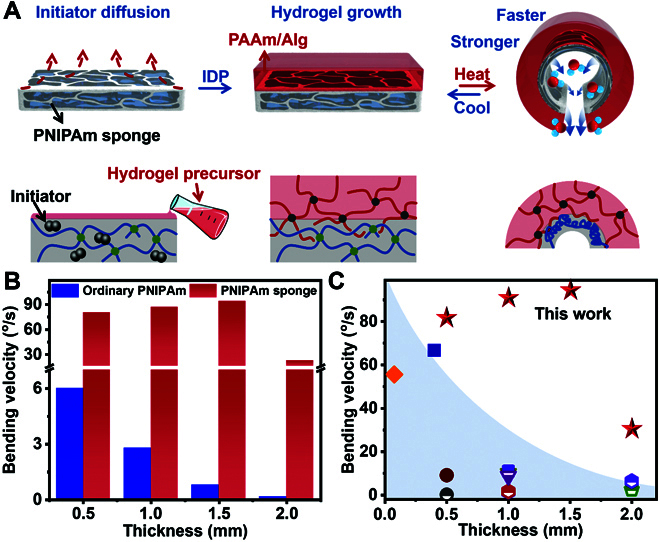
The fabrication and performance of the thermoresponsive hydrogel actuator. (A) The IDP-induced hydrogel growing process. The thermoresponsive hydrogel actuator was prepared by pouring the hydrogel precursor onto the surface of the PNIPAm sponge containing the initiator. (B) The actuating velocity of the bilayer hydrogel actuator with different thicknesses of the ordinary PNIPAm layer and the PNIPAm sponge layer. (C) Comparison of bending velocity of the existing hydrogel actuator [29,39–41,45–53].

Living organisms can generally generate complex deformation based on their anisotropic structure [39]. Various anisotropic structures such as bilayer, gradient, orientated, and patterned are proposed and introduced into hydrogel networks during the polymerization to replicate these biological behaviors [40–43]. However, limited by the unique fabrication process of the PNIPAm sponge, it is not easy to prepare an anisotropic PNIPAm sponge. Recently, we proposed a universal strategy to grow a passive hydrogel layer on the surface of the as-prepared hydrogels via IDP [44]. Firstly, as shown in Fig. 2A, the as-prepared PNIPAm sponge was immersed in the initiator solution (APS). After wiping the surface, a hydrogel precursor was poured onto the surface. With the diffusion of the initiator from the PNIPAm sponge to the hydrogel precursor, a new hydrogel layer could grow and firmly anchor to the PNIPAm sponge via an interpenetrating network. Therefore, the composite hydrogel could exhibit reversible and fast bending deformation in response to external change (heat and cool).

As mentioned above, increasing the thickness of the active hydrogel layer could increase the exported energy, but the increased thickness could also influence the actuating property. When the thickness of the ordinary hydrogel increased from 0.5 to 2.0 mm, the maximum bending angle would decrease to 100°, and the actuating velocity also decreased from 6.01°/s to 0.14°/s. Similarly, when the PNIPAm sponge replaced the ordinary hydrogel, although the maximum bending angle would also influence the thickness, the maximum bending angle could only decrease to 256° (Fig. S6). Compared to the ordinary hydrogel, the actuating velocity of the PNIPAm sponge-based bilayer hydrogel actuator would increase about one order of magnitude (Fig. 2B). It was worth noting that due to the fast water exchange property, when the thickness of the PNIPAm sponge layer increased in a low range, the PNIPAm sponge could exhibit a stable fast swelling/deswelling property. However, owing to the mechanical limit of the passive hydrogel layer, the thinner PNIPAm sponge layer was unable to actuate the deformation of the whole bilayer hydrogel. Thus, the actuating velocity of the PNIPAm sponge-based bilayer hydrogel actuator would slightly increase from 0.5 to 1.5 mm with the increase in thickness and decreased at 2-mm thickness because of the excessive volume. Horizontally compared with the existing hydrogel actuators, the bending velocity of the PNIPAm sponge-based hydrogel actuator is fast within the universal thickness range (Fig. 2C). This phenomenon indicated that improving the exported energy by increasing thickness without impairing the actuating velocity was feasible. Besides, the actuating velocity was also adjustable owing to the positive relationship with the external temperature. With the increase of the external temperature from 30 to 50 °C, the actuating velocity would increase from 0°/s to 88°/s. It is worth noting that benefiting from the excellent actuating property of the PNIPAm sponge, the actuating velocity of the PNIPAm sponge-based bilayer hydrogel actuator could also reach 15°/s at 34 °C, which was closed to the phase transition temperature of PNIPAm chains (~32 °C) (Fig. S7).

### Programmable evolution of the PNIPAm sponge from isotropic to anisotropic

Utilizing IDP as a hydrogel evolution strategy, the as-prepared PNIPAm sponge could be endowed with anisotropic structure and new functions. For example, when Fe_3_O_4_ nanoparticles were introduced into the hydrogel precursor, the photothermal responsive hydrogel layer could grow from the surface of PNIPAm sponges via IDP. Owing to the excellent photothermal effect of Fe_3_O_4_ nanoparticles, when a bench of NIR is locally irradiated on the surface of the upper hydrogel layer, the temperature of the upper hydrogel layer could increase to about 65 °C under 8 W NIR while that of the bottom hydrogel layer without Fe_3_O_4_ could only rise to about 35 °C (Fig. S8). In addition, the generated heat would flow along the thickness direction, triggering the thermoresponsive deformation of the bottom hydrogel layer (Fig. S9). Therefore, the deformation of the photothermal bilayer hydrogel could be controlled by the power and irradiate the position of NIR (Fig. S10).

**Fig. 3.**
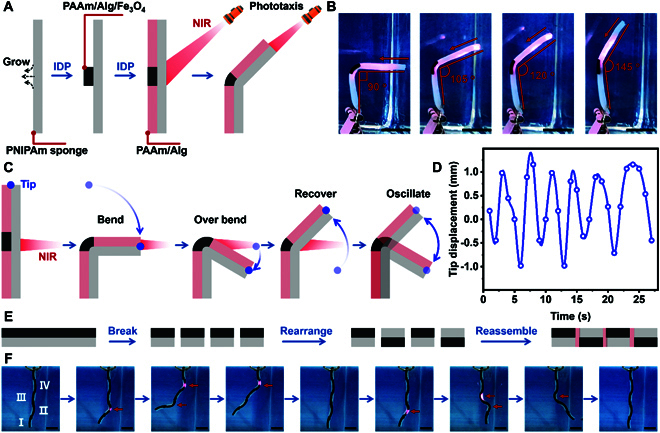
Evolution-based multifunctional hydrogel actuator. (A) Illustration schematic showing the preparation of the hydrogel oscillator. The photothermal hydrogel oscillator was fabricated via a growing photothermal hydrogel layer containing Fe_3_O_4_ in the middle area and a passive hydrogel layer in the rest area, respectively. (B) Images show the phototaxis of the hydrogel actuator. (C) Illustration schematic showing the oscillating mechanism of the hydrogel oscillator. The hydrogel oscillator would bend toward the direction of the NIR source. (D) Tip trajectory of the hydrogel oscillator during the oscillating process. (E) Illustration schematic showing the fabricating process of the hydrogel actuator with a multisection structure. A novel hydrogel actuator with a multisection structure was fabricated by cutting a bilayer hydrogel actuator into 4 sections and rearranging them via IDP. (F) Images show the multidimensional deformation of the hydrogel actuator with a multisection structure with the assistance of programmable NIR. Scale bars: 1 cm.

Moreover, the as-prepared PNIPAm sponge could evolve to more deformation modes by selectively growing the photothermal hydrogel layer and passive hydrogel layer. For instance, as shown in Fig. 3A, a photothermal hydrogel layer grew in the middle area, and the passive hydrogel layer grew on the rest area of the surface of the PNIPAm sponge strip via IDP. Subsequently, when NIR is irradiated onto the photothermal hydrogel layer, plenty of heat would be generated since the photothermal effect of Fe_3_O_4_ nanoparticles triggered the shrinkage of the PNIPAm sponge and actuated the whole hydrogel strip to the direction of the NIR source. With the bending of the entire hydrogel strip, the top part would hinder part of NIR light, which decreases the light power irradiated on the photothermal hydrogel layer. Thus, the hydrogel strip would exhibit phototaxis and be directed to any direction of the NIR source from 90° to 145° (Fig. 3B).

Interestingly, benefiting from the fast actuation of the PNIPAm sponge, the top part of the hydrogel strip would bend over the balance line and blanket the NIR, which caused the temperature of the photothermal hydrogel layer to quickly decrease and further induce the shape recovery of the PNIPAm sponge. During the shape recovery process, the top part would recover over the balance line, exposing the photothermal hydrogel layer to NIR, beginning the next cycle (Fig. 3C). Therefore, the hydrogel strip could exhibit oscillatory behavior with an amplitude of 2 mm and a period of 5 s (Fig. 3D).

According to the description above, the IDP strategy can evolve the hydrogels with isotropic deformation property to hydrogel actuators with anisotropic deformation, further developing oscillating behavior. Moreover, even the evolved hydrogel actuator could also be secondly evolved to adapt to the changing demand of the external environment. For instance, an isotropic PNIPAm sponge could obtain a bilayer structure and exhibit bending deformation after growing the photothermal hydrogel layer containing Fe_3_O_4_. Subsequently, the bilayer hydrogel would be cut into 2 blocks and immersed in the APS solution. After flipping one of the blocks and rearranging the 2 cutting blocks, the hydrogel precursor was injected into the cavity between the 2 hydrogel blocks. Owing to osmotic pressure, APS would diffuse from the hydrogel blocks to the hydrogel precursor while the monomer would diffuse from the hydrogel precursor to the hydrogel blocks, leading to the polymerization in the interface between the hydrogel blocks and the hydrogel precursor. Thus, the 2 hydrogel blocks could be reconnected via the physical entanglements between the new hydrogel network and the original hydrogel network. Due to the anisotropic structure existing in both horizontal and vertical direction, the new bilayer hydrogel could generate more complex deformation from strip to “S” shape in hot water or under NIR (Fig. S11). Besides, even if the hydrogel was cut into more blocks, they could be effectively reconnected similarly (Fig. 3E). Based on the arthropod-inspired multisection structure, the new anisotropic hydrogel actuator could exhibit a higher degree of freedom. As shown in Fig. 3F, when a NIR is irradiated onto the part II, the new hydrogel actuator would bend like the regular bilayer hydrogel. Subsequently, adding a new NIR irradiating onto the part IV would secondarily bend to a “U” shape. Therefore, by programmatically regulating the NIR, the new hydrogel actuator could generate 8 types of configurations and multiple degrees of freedom in the 2D direction.

### Inchworm-inspired locomotion via the dynamic mortise-and-tenon interlock

**Fig. 4.**
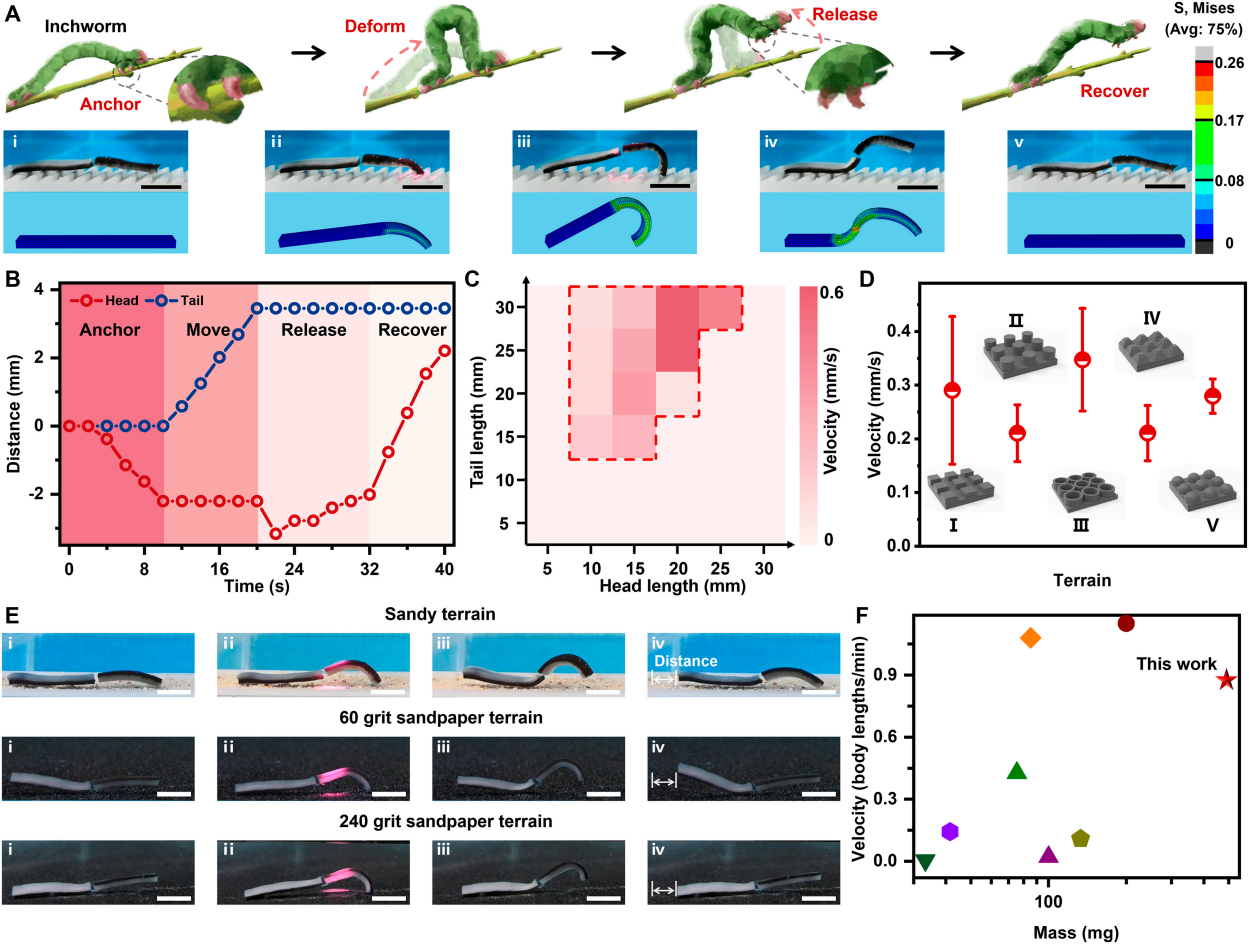
Inchworm-inspired locomotion of the hydrogel actuator. (A) The inchworm-inspired locomotion of the bilayer hydrogel actuator. The hydrogel actuator is anchored to the substrate via deformation, inducing mortise-and-tenon interlock and crawling forward. Then, the body would deform to an “S” shape, removing the mortise-and-tenon interlock and beginning the next cycle. (B) The feature trajectories of the head and tail of the hydrogel actuator within one crawling cycle. (C) The crawling velocity of the bilayer hydrogel actuator with different ratios of head and tail. (D and E) The locomotion velocity and process of the hydrogel actuator in different terrains. (F) Comparison of moving velocity of the existing soft robots[19,20,22,27,48,54,55]. Scale bars: 1 cm.

Based on the evolution, the new bilayer hydrogel actuator was capable of transferring the in situ deformation to locomotion by imitating the crawling mode of the inchworm. As shown in Fig. 4A, the inchworm, an arthropod with nonconstricted body segments, can crawl via wave-like contractions of innervated muscles. Similarly, the reconfigured bilayer hydrogel could also realize this biomimetic locomotion with the assistance of programmed NIR in the following steps: (a) The head's anterior section of the bilayer hydrogel was exposed under NIR and generated bending deformation due to the thermoresponsive shrinkage of the PNIPAm sponge, firmly anchoring with the ratchet-shaped substrate via the mortise-and-tenon interlock effect. (b) With the movement of the NIR source from anterior to posterior, the significant body of the bilayer hydrogel would move forward. (c) Simultaneously, the middle section deformed to an “S” shape while the anterior section gradually recovered, inducing the release of the mortise-and-tenon interlock. (d) After removing the NIR source, the bilayer hydrogel completely recovered to the initial shape, beginning the next cycle. Subsequently, the shape deformation statement within every motion step was simulated by finite element modeling, and the results were highly consistent with the experimental results. Also, the motion track of the head and tail was recorded, showing the feature trajectories of every step (Fig. 4B). Repeating the cycle, the bilayer hydrogels moved forward to 18 mm within 160 s (Fig. S12). Moreover, the moving speed could be improved by adjusting the ratio of head and tail, and the maximum could reach 0.6 mm/s (Fig. 4C and Movie S2). It is worth noting that adopting a soft deformation-induced dynamic mortise-and-tenon interlock improved the environment adaptability. Thus, the bilayer hydrogel could adapt and quickly crawl above the various surfaces with bumps of different shapes (Fig. 4D, Fig. S13, and Movie S3). Similarly, as shown in Fig. 4E, the bilayer hydrogel could also crawl above the ordinary sandy terrain. Even when the degree of roughness decreased from 60 grit to 240 grit, the crawling mechanism was still appropriate, and the bilayer hydrogel would move ~6.5 mm in one cycle (Fig. S14).

In general, decreasing the volume and weight of a soft robot to increase the moving speed is a common and efficient strategy. It is worth noting that increasing the volume means an individual soft robot could export more mechanical energy. Thus, horizontally compared with the reported hydrogel-based soft robot, this system could exhibit a faster-moving rate even with a heavier body owing to the excellent photothermal effect of Fe_3_O_4_ and the thermoresponsiveness of the PNIPAm sponge (Fig. 4F). Although there is still a huge gap between hydrogel-based soft robots and other soft robots in terms of moving speed, considered the special actuating mechanism of stimuli-responsive hydrogel, it is still considerable with the existing hydrogel-based soft robots [56,57]. In fact, benefiting from the larger volume, the actual moving speed of the bilayer hydrogel actuators was the fastest. Moreover, these bilayer hydrogel actuators could also be utilized as hydrogel motors owing to their powerful energy. As shown in Fig. S15, the bilayer hydrogel actuator was connected to a 2-g cargo via DIP and drives it with an instantaneous speed of 3.6 mm/s or drives a 12-g cargo with a high speed of about 0.5 mm/s. In addition, the adaptive deformation of the bilayer hydrogel actuator also allows the hydrogel motor to crawl on a slope of 5° and10° loading or nonloading static cargo (Fig. S16).

### Controllable 2-way and 2D locomotion

According to the above results, by utilizing the IDP strategy to reconfigure the anisotropic structure, the as-prepared PNIPAm sponge could be endowed with unidirectional locomotion above rough terrains. Thus, the above hydrogel actuator with a head–tail structure could be further reconfigured again, in order to evolve to more efficient locomotion. For example, the hydrogel actuator with a head–tail structure was cut into 2 blocks again and reconnected to form a new hydrogel actuator with a head–tail–head structure in the same way. Benefiting from this new structure, the new hydrogel actuator could obtain a new locomotion mode, which is the rear drive mode. As shown in Fig. S17, the posterior section of the hydrogel actuator would firstly deform to an “S” shape and anchored onto the surface of the ratchet-shaped substrate. With the recovery process of the hydrogel actuator, a forward force was generated, pushing the whole hydrogel to crawl forward. It is worth noting that when the 2 heads of the hydrogel actuator exported the same drive mode, it could exhibit 2-way locomotion by controlling the NIR (Fig. S18).

**Fig. 5.**
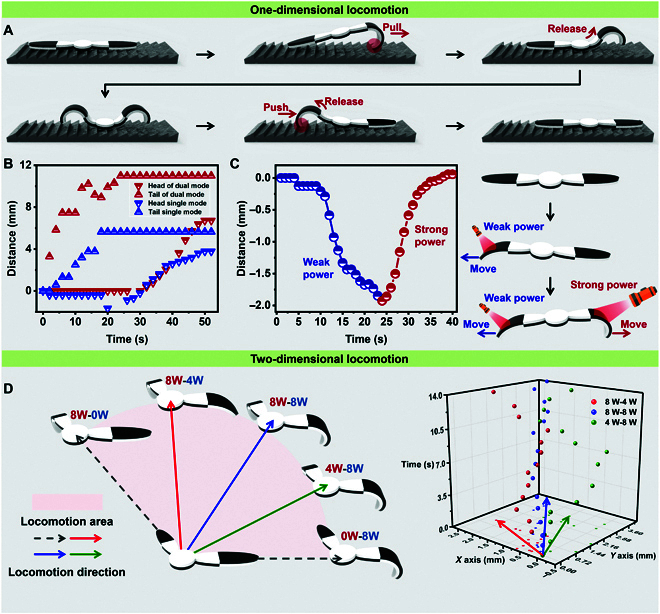
The multimode locomotion of the bilayer hydrogel actuator. (A) Schematic showing the dual crawling mode where both the head and tail of the hydrogel actuator could export forward force. (B) The feature trajectories of the head and tail of the hydrogel actuator in dual crawling mode. (C) The bilayer hydrogel actuator tended to crawl to the side with stronger NIR when a weak NIR and stronger NIR are irradiated successively. (D) The 2D locomotion of the bilayer hydrogel actuator within the included angle between the left and right arm.

Besides, if one side of the hydrogel actuator generated a pull force while another generated a push force, the hydrogel actuator could export more forward energy (Fig. 5A). Compared to the single drive mode, by utilizing the dual drive mode, the hydrogel actuator could span longer distances in one crawling cycle (Fig. 5B and Movie S4). Furthermore, when a weak NIR (4 W) is irradiated on the left, the new hydrogel actuator would gradually crawl to the left. Then, a competitive NIR (8W) is irradiated on the right, generating a stronger pull than the left. Thus, the new hydrogel actuator would be pulled to the original point (Fig. 5C). Moreover, when a weak NIR and a strong NIR are simultaneously irradiated on the left and right, the asymmetrical pull is generated, and the new hydrogel actuator tends to crawl toward the side with stronger NIR (Fig. S19).

These phenomena indicated that coupling both sides of pull or push forces could control the crawl direction and speed of the hydrogel actuator. To test this, the included angle between the left and right arm of the new hydrogel actuator was decreased to 120°, and 2 NIR with different powers were simultaneously irradiated on the left and right arm. As shown in Fig. 5D, the new hydrogel actuator would crawl in the same direction as the 2 forces coupled direction, indicating that by changing the power of NIR, the hydrogel actuator would freely crawl within the range of the included angle of the 2 arms (Movie S5).

### Potential applications of hydrated soft robots with off-road capability

**Fig. 6.**
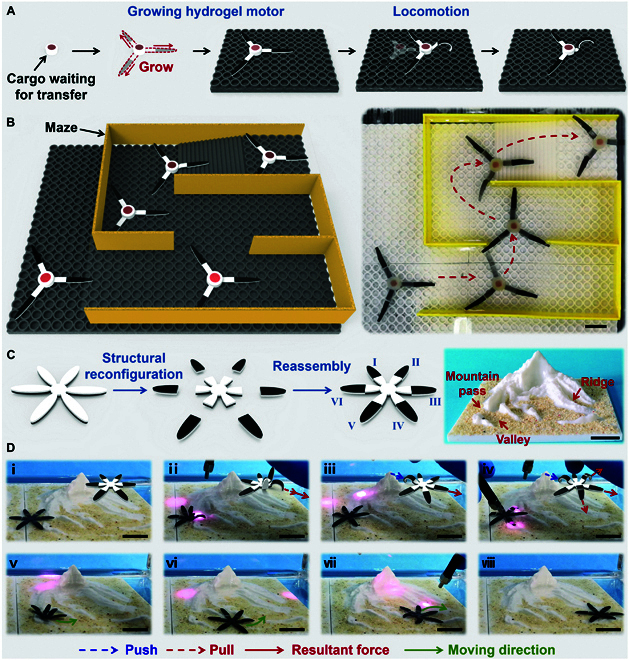
Two-dimensional off-road locomotion and applications of bilayer hydrogel motors. (A) Schematic showing the assembly process of the hydrogel motor. The model component was loaded into the PNIPAm sponge and assembled with 3 bilayer hydrogel motors via IDP. (B) The composite soft robot could crawl and pass through a 2D maze. (C) The fabrication process of the off-road hydrogel robot. A 6-claw-shaped bilayer hydrogel was cut into 7 parts and reconnected via IDP. (D) Off-road process of the hydrogel robot. The reconfigured 6-claw-shaped hydrogel actuator could pass through the narrow mountain pass and off-road the complex 2D terrain via the coupling deformation of 6 claws. Scale bars: 2 cm.

Benefiting from the efficient and universal hydrogel assemble strategy, IDP, the as-prepared PNIPAm sponge could evolve from an isotropic to an anisotropic structure, from a strip to a 2D shape, realizing 2D locomotion. Herein, we demonstrated its potential application in soft robots, cargo transportation, and biomimetic devices. Figure 6A shows that the 3 abovementioned bilayer hydrogel actuators were applied as hydrogel motors and assembled to static PNIPAm sponge via IDP. When the 3-arm soft robot crawled into a confined environment, it could decrease its volume via the deformation of the arms to adapt and pass through the narrow passage (Fig. S20). Furthermore, through reasonable and efficient cooperation of the 3 arms, the 3-arm soft robot could crawl, turn direction, and even climb stairs, eventually escaping the confined environment (Fig. 6B and Movie S6). Therefore, the static PNIPAm sponge could be completely transported to the destination after physically removing the hydrogel motor.

Because of the powerful energy export capability, the PNIPAm sponge could also be utilized to fabricate the mechanically discolorable device. Four hydrogel actuators were assembled into a cylindrical PNIPAm sponge based on the abovementioned method. With the assistance of NIR, the cylindrical PNIPAm sponge could move on a preset track (Fig. S21). For example, according to the command (go to the right), the NIR would irradiate to the hydrogel actuator on the right, inducing the locomotion of the hydrogel actuator and pulling the cylindrical PNIPAm sponge to the right. Similarly, when the NIR is irradiated to the hydrogel actuator on the left, the cylindrical PNIPAm sponge would be pulled to the initial position or the left area. Thus, by adjusting the 4 hydrogel actuators' locomotion, the cylindrical PNIPAm sponge could freely move within the central area.

Furthermore, to increase the visibility, the command was transferred as a fluorescent signal by growing a multicolor fluorescent hydrogel on the surface of the PNIPAm sponge. After assembling the 4-arm soft robot with a black shell, the mechanically discolorable device was obtained (Fig. S22). In the initial situation, the PNIPAm sponge stopped in the central part and exported a red fluorescent signal through the small hole of the shell. When a NIR is irradiated on the right of the hydrogel actuator, the whole PNIPAm sponge is pulled to the right, exposing the blue fluorescent signal with the left arrow. Similarly, the fluorescent signal would turn back by irradiating the left hydrogel actuator. Utilizing the 2D locomotion of the PNIPAm sponge, the mechanically discolorable device exhibited a high fluorescent signal.

As discussed above, given the powerful energy export capability of the PNIPAm sponge, by increasing the number of tentacles, the new hydrogel actuator could further obtain freely off-road locomotion ability on the 2D complex terrains. As shown in Fig. 6C, the anisotropic structure of the 6-claw-shaped hydrogel was reconfigured and reconnected via IDP. With the assistance of programmed NIR, the 6-claw-shaped hydrogel could freely crawl toward the direction of the NIR source (Fig. S23), realizing 2D locomotion. Besides, due to the high degree of deformation freedom of each tentacle, the 6-claw-shaped hydrogel could adapt to the complex sandy terrain and freely crawl (Fig. S24). In addition, when a huge obstacle was on the way of the hydrogel crawls, tentacles II and IV would bend to decrease the whole volume. Thus, a half-body of the hydrogel could pass through the narrow mountain pass by the programmed deformation of tentacle III. Subsequently, upon the dual drive mode as mentioned above, tentacle VI would generate a push force while tentacles II and IV generate a pull force. Thus, a powerful resultant force was generated, pulling the entire hydrogel across the narrow mountain pass (Fig. 6D). Similarly, by coupling different tentacles, the 6-claw-shaped hydrogel could change both its body volume and crawling direction and eventually overstep and pass through 3D complex terrains, such as ridges and valleys, to reach the destination (Movie S8).

## Discussion

In conclusion, we have demonstrated the design principles and control strategy to realize adaptive deformation and off-road locomotion for hydrated soft robots via the deformation-induced dynamic mortise-and-tenon interlock. In this system, inspired by the self-growth and evolution of living organisms, a photothermal hydrogel layer grew from the surface of the as-prepared isotropic PNIPAm sponge to form an anisotropic bilayer structure via IDP. Moreover, the anisotropic configurations of the bilayer hydrogel could be reconfigured and reassembled to adapt to the change in external requirements, exhibiting multiple degrees of deformation freedom and various morphologies with the assistance of programmed NIR. Based on adaptive deformation, a mortise-and-tenon interlock could be dynamically formed when the hydrogel actuator bent or recovered, which generated periodic propulsion, endowing the hydrogel actuator with off-road locomotion. Interestingly, benefiting from the powerful mechanical energy export capability of the PNIPAm sponge, the hydrogel actuator could be utilized as a motor to move a cargo several times larger than itself. Therefore, after assembling several hydrogel motors, even a static cargo could be activated and crawl above the 2D rough substrate or overstep complex sandy terrains. This strategy is beneficial for designing and fabricating the soft robot and may attract attention from the relative fields of deformable materials, cargo transfer, and signal devices.

## Materials and Methods

### Materials

Acrylamide was purchased from Shanghai Sinopharm Chemical Reagent Co., Ltd.; NIPAm, sodium alginate, APS, BIS, TEMED, Fe_3_O_4_ nanoparticles, and rhodamine B were obtained from Aladdin Shanghai Reagent Co. Ltd.

All the chemicals were used as received.

### Instruments

The tensile and compression tests were performed on a CMT-1104 universal testing machine (CMT-1104, SUST Electrical Equipment Co.). The maximum output force of the PNIPAm sponge was measured by an electronic balance (METTLER TOLEDO ME204). The photothermal test was measured by an NIR laser source with a wavelength of 808 nm (BWT Beijing, K808DAHFN-15.00 W). The temperature is measured by an infrared camera (Optris PI-450i purchased from Optris GmBH).

### Preparation of the PNIPAm sponge

The PNIPAm sponge was fabricated according to our previous work. In brief, 2 g of NIPAm monomers, 20 mg of BIS, and 20 mg of APS were dissolved 10 ml of deionized water to obtain a clear solution. Subsequently, 20 μl of TEMED was added to the above solution, and it was quickly poured into a self-made mold with a thickness of 1 mm. The hydrogel precursor must be rapidly placed at −30 °C for 0.5 h to form ice crystals. When the hydrogel precursor was completely frozen, the polymerization temperature was set to -5 °C and the precursor was further polymerized for 12 h. Finally, the PNIPAm sponge was obtained after immersing it in water for 24 h to remove the unreacted monomers.

### Preparation of the bilayer hydrogel actuator

According to our previous work, the bilayer hydrogel was fabricated via IDP. In brief, the as-prepared PNIPAm sponge was immersed in a 15 mg/ml APS solution. Then, the hydrogel precursor containing 710 mg of acrylamide, 21.3 mg of BIS, 150 μl of TEMED, and 10 ml of sodium alginate (2%) was poured onto the surface of the treated PNIPAm sponge. A self-made hollow mold was utilized within the hydrogel growth process to limit the total thickness to 2 mm. Finally, the bilayer hydrogel was transferred to a 0.1 M Ca^2+^ solution for 5 min to stabilize the bilayer structure.

### Structural reconfiguration of the bilayer hydrogel actuator

The as-prepared bilayer hydrogel was cut into 2 blocks and immersed into a 15 mg/ml APS solution for 5 min respectively. Then, the 2 hydrogel blocks were arranged and the hydrogel precursor was poured into the gap between the 2 hydrogel blocks. After 5 min, the 2 hydrogel blocks would be connected via IDP.

### Preparation of the mechanical discoloration device

The as-prepared PNIPAm sponge was first immersed in a 15 mg/ml APS solution for 5 min. Then, a self-made PDMS mold with a round hole was covered on the surface of the PNIPAm sponge, and the red fluorescent hydrogel precursor containing rhodamine B as the fluorescent group was poured into the hole of the PDMS mold. After waiting for 5 min, the red fluorescent hydrogel would grow on the surface of the PNIPAm sponge and then the unreacted hydrogel precusor was washed. Similarly, the blue fluorescence hydrogel containing polyacrylamide-co-1-pyrenylmethyl acrylate and the yellow-green fluorescence hydrogel containing polyacrylamide-co-4-(*N*,*N*-dimethylaminoethylene)amino-*N*-allyl-1,8-naphthalimide were prepared in the same way. Finally, the mechanical discoloration device was obtained by connecting 4 bilayer hydrogel actuators to the body of the PNIPAm sponge via IDP.

## Data Availability

All data are available in the main text or the Supplementary Materials.

## Supplementary Materials

Supplementary 1 Fig. S1. Fabrication process of the PNIPAm sponge. Fig. S2. Shrinking property of the PNIPAm sponge and the ordinary PNIPAm. Fig. S3. Water absorption capacity of the PNIPAm sponge and the ordinary PNIPAm hydrogel. Fig. S4. Hydrogel wash process of the PNIPAm sponge and the ordinary PNIPAm hydrogel. Fig. S5. Muscle-inspired energy output process of the PNIPAm sponge. Fig. S6. Variation of bending angles of the PNIPAm sponge and the ordinary PNIPAm hydrogel. Fig. S7. Actuating velocity of the PNIPAm sponge. Fig. S8. Photothermal curve of Fe_3_O_4_ NPs containing hydrogel. Fig. S9. The heat flow condition of the hydrogel. Fig. S10. Photothermal responsive deformation process. Fig. S11. Structural reconfiguration of hydrogel. Fig. S12. Feature trajectories of crawling cycles. Fig. S13. Images of different landforms. Fig. S14. Locomotion process of the hydrogel actuator above different sandpaper terrains. Fig. S15. Energy export capability of the PNIPAm sponge. Fig. S16. The hydrogel motor crawled on a slope loading or nonloading the cargo. Fig. S17. Feature trajectories of the hydrogel actuator in rear drive mode. Fig. S18. The 2-way crawling of the bilayer hydrogel actuator. Fig. S19. The competitive crawling of the bilayer hydrogel actuator. Fig. S20. Crawl process of the composite soft robot passing through a narrow passage. Fig. S21. Moving process of static cargo loaded with hydrogel motors. Fig. S22. Fabrication and discoloration of the mechanical discoloration device. Fig. S23. Free locomotion of the bilayer hydrogel actuator. Fig. S24. Off-road locomotion of the bilayer hydrogel actuator.

Supplementary 2 Movie S1. Thermoresponsiveness of the PNIPAm sponge.

Supplementary 3 Movie S2. Inchworm-inspired locomotion of the hydrogel actuator.

Supplementary 4 Movie S3. 1D locomotion of the hydrogel actuator above different terrains.

Supplementary 5 Movie S4. Multimode of locomotion.

Supplementary 6 Movie S5. 2D locomotion of the hydrogel actuator.

Supplementary 7 Movie S6. Hydrogel motor moved the static cargo passing through the maze.

Supplementary 8 Movie S7. 2D free crawl of the hydrogel actuator.

Supplementary 9 Movie S8. 2D off-road locomotion of the hydrogel actuator.
