# Supplementary material for: The Dynamic Mortise-and-Tenon Interlock Assists Hydrated Soft Robots Toward Off-Road Locomotion
Source: Research (Wash D C). 2022 Dec 19;2022:0015. doi: 10.34133/research.0015 (PMC11407522; doi:10.34133/research.0015)
Supplement: Supplementary 1 — Fig. S1. Fabrication process of the PNIPAm sponge. Fig. S2. Shrinking property of the PNIPAm sponge and the ordinary PNIPAm. Fig. S3. Water absorption capacity of the PNIPAm sponge and the ordinary PNIPAm hydrogel. Fig. S4. Hydrogel wash process of the PNIPAm sponge and the ordinary PNIPAm hydrogel. Fig. S5. Muscle-inspired energy output process of the PNIPAm sponge. Fig. S6. Variation of bending angles of the PNIPAm sponge and the ordinary PNIPAm hydrogel. Fig. S7. Actuating velocity of the PNIPAm sponge. Fig. S8. Photothermal curve of Fe3O4 NPs containing hydrogel. Fig. S9. The heat flow condition of the hydrogel. Fig. S10. Photothermal responsive deformation process. Fig. S11. Structural reconfiguration of hydrogel. Fig. S12. Feature trajectories of crawling cycles. Fig. S13. Images of different landforms. Fig. S14. Locomotion process of the hydrogel actuator above different sandpaper terrains. Fig. S15. Energy export capability of the PNIPAm sponge. Fig. S16. The hydrogel motor crawled on a slope loading or nonloading the cargo. Fig. S17. Feature trajectories of the hydrogel actuator in rear drive mode. Fig. S18. The 2-way crawling of the bilayer hydrogel actuator. Fig. S19. The competitive crawling of the bilayer hydrogel actuator. Fig. S20. Crawl process of the composite soft robot passing through a narrow passage. Fig. S21. Moving process of static cargo loaded with hydrogel motors. Fig. S22. Fabrication and discoloration of the mechanical discoloration device. Fig. S23. Free locomotion of the bilayer hydrogel actuator. Fig. S24. Off-road locomotion of the bilayer hydrogel actuator. [file research.0015.f1.docx]

**Dynamic mortise-and-tenon interlock assist hydrated soft robot toward off-road locomotion**

Baoyi Wu^1,2^, Yaoting Xue^3^, Israt Ali^4^, Huanhuan Lu^5^, Yuming Yang^6^, Xuxu Yang^3^, Wei Lu^1,2^ Yinfei Zheng^6^* and Tao Chen^1,2^*

^1^ Key Laboratory of Marine Materials and Related Technologies, Zhejiang Key Laboratory of Marine Materials and Protective Technologies, Ningbo Institute of Material Technology and Engineering, Chinese Academy of Sciences, Ningbo, 315201, China.

^2^ School of Chemical Sciences, University of Chinese Academy of Sciences, 19A Yuquan Road, Beijing 100049, China.

^3^ Department of Engineering Mechanics, Zhejiang University, Hangzhou 310027, China.

^4^ INRS-EMT, 1650 Boul. Lionel Boulet, Varennes, J3X 0A1, Canada.

^5^ College of Chemical Engineering, Ningbo Polytechnic, Ningbo, 315800, China.

^6^ College of Biomedical Engineering and Instrument Science, Key Laboratory for Biomedical Engineering of Ministry of Education Ministry of China, Zhejiang University, Hangzhou 310027, China

E-mail: zyfnjupt@zju.edu.cn, tao.chen@nimte.ac.cn

Keywords: Soft robots, biomimetic device, All-terrain locomotion, Hydrogel motor, Interfacial diffusion polymerization

**Contents**

**Figures S1.** Fabrication process of PNIPAm sponge. 1

**Figures S2.** Shrinking property of PNIPAm sponge and ordinary PNIPAm 1

**Figures S3.** Water absorption capacity of PNIPAm sponge and ordinary PNIPAm hydrogel. 1

**Figures S4.** Hydrogel wash process of PNIPAm sponge and ordinary PNIPAm hydrogel. 1

**Figures S5.** Muscle-inspired energy output process of PNIPAm sponge. 2

**Figures S6.** Variation of bending angles of PNIPAm sponge and ordinary PNIPAm hydrogel. 2

**Figures S7.** Actuating velocity of PNIPAm sponge. 3

**Figures S8.** Photothermal curve of Fe_3_O_4_ NPs containing hydrogel. 4

**Figures S9.** The heat flow condition of the hydrogel 3

**Figures S10.** Photothermal responsive deformation process. 4

**Figures S11.** Structural reconfiguration of hydrogel. 4

**Figures S12.** Feature trajectories of crawling cycles. 5

**Figures S13.** Images of different landform. 5

**Figures S14.** The locomotion process of hydrogel actuator above different sandpaper terrains. 5

**Figures S15.** Energy export capability of PNIPAm sponge. 6

**Figures S16.** The hydrogel motor crawled on a slope loading or non-loading the object. 6

**Figures S17.** The feature trajectories of hydrogel actuator in rear drive mode. 7

**Figures S18.** The two-way crawling of the bilayer hydrogel actuator. 7

**Figures S19.** The competitive crawling of bilayer hydrogel actuator. 8

**Figures S20.** Crawl process of the composite soft robot passes through narrow passage. 8

**Figures S21.** Moving process of static object loaded with hydrogel motors. 9

**Figures S22.** Fabrication and discoloration of mechanical discoloration device 9

**Figures S23.** Freely locomotion of bilayer hydrogel actuator. 10

**Figures S24.** Off-road locomotion of bilayer hydrogel actuator. 10

Supplementary Movie 10


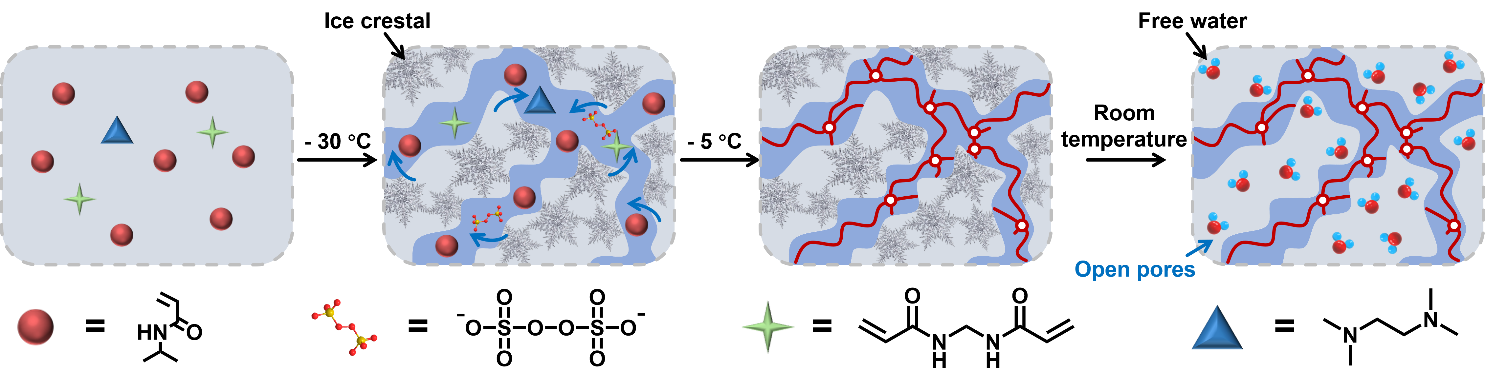


**Figure S1.** Illustration show the fabrication process of PNIPAm sponge.


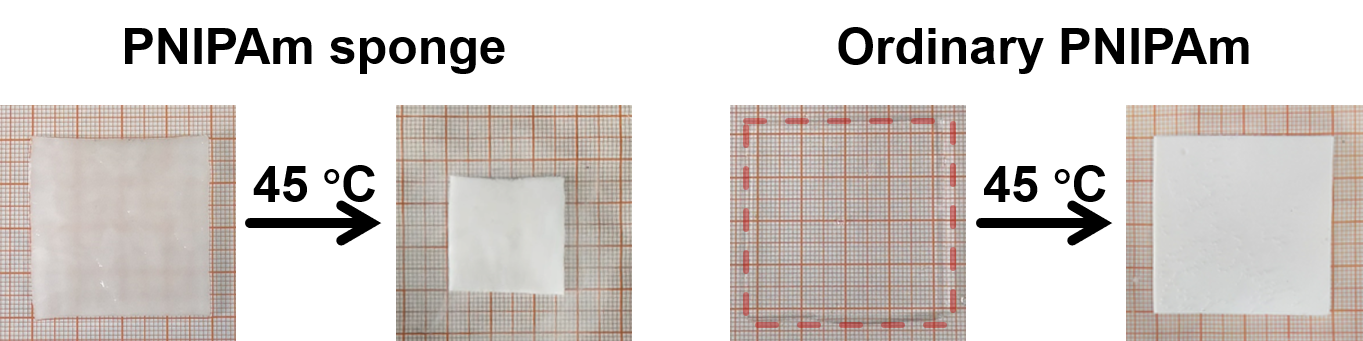


**Figure S2.** Images shown the shrinking property of PNIPAm sponge and ordinary PNIPAm, respectively.


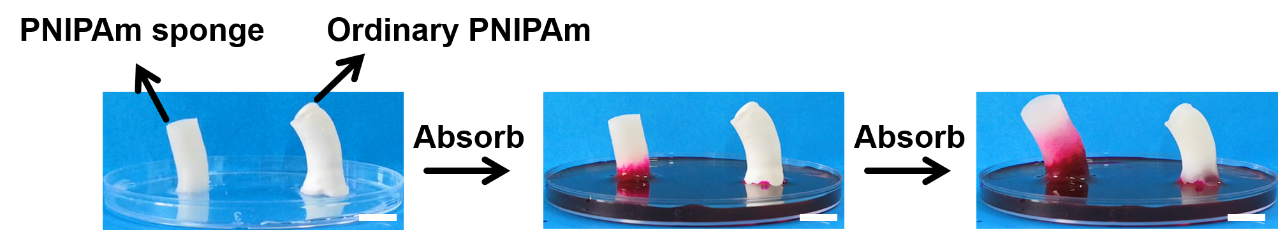


**Figure S3.** Images shown the water absorption capacity of PNIPAm sponge and ordinary PNIPAm respectively.


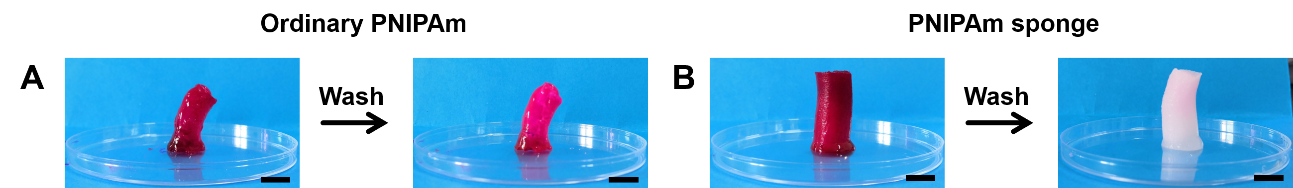


**Figure S4.** Images shown the hydrogel wash process where the red dye in ordinary PNIPAm could not be washed (A) while the PNIPAm sponge could (B). Scale bars: 1 cm.


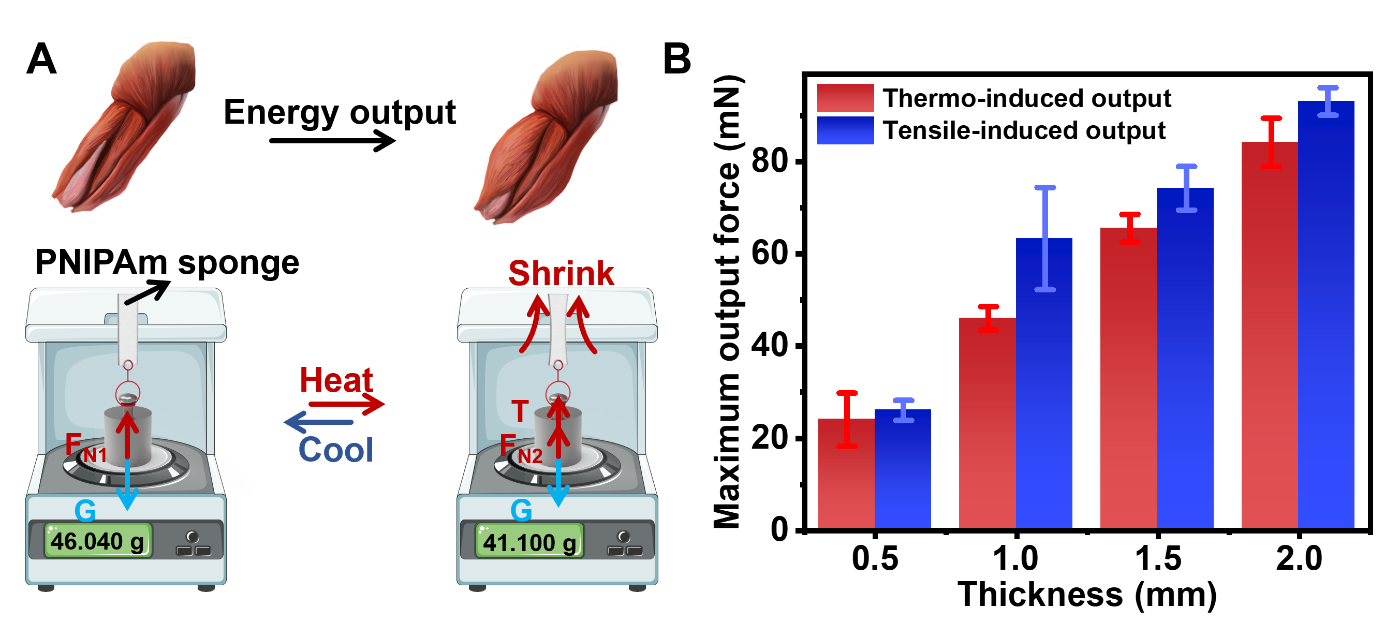


**Figure S5.** A) Illustration schematic the muscle-inspired energy output process and the corresponding measurement method. B) The maximum output force of PNIPAm sponge with different thickness in thermo-induced and tensile-induced mode respectively.


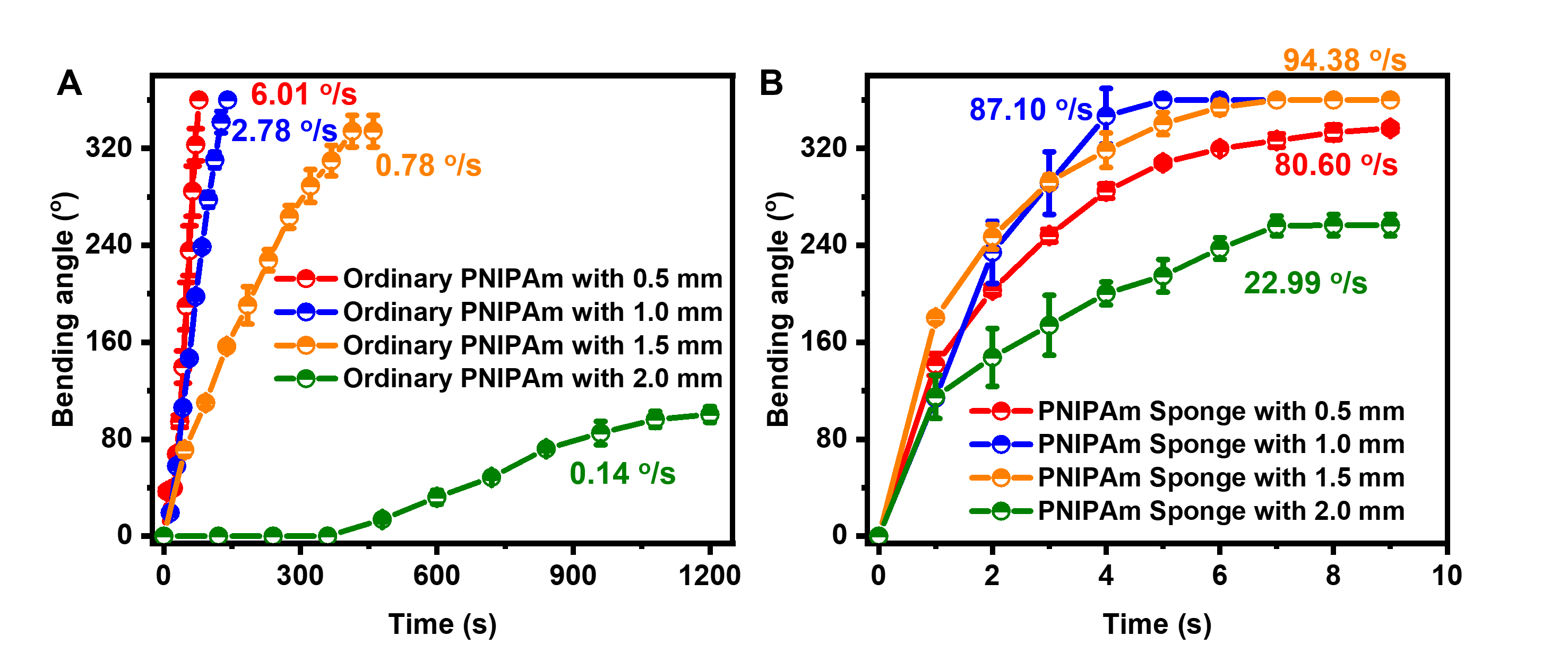


**Figure S6.** Variation of bending angles of the (A) ordinary PNIPAm and PNIPAm sponge with different thickness.


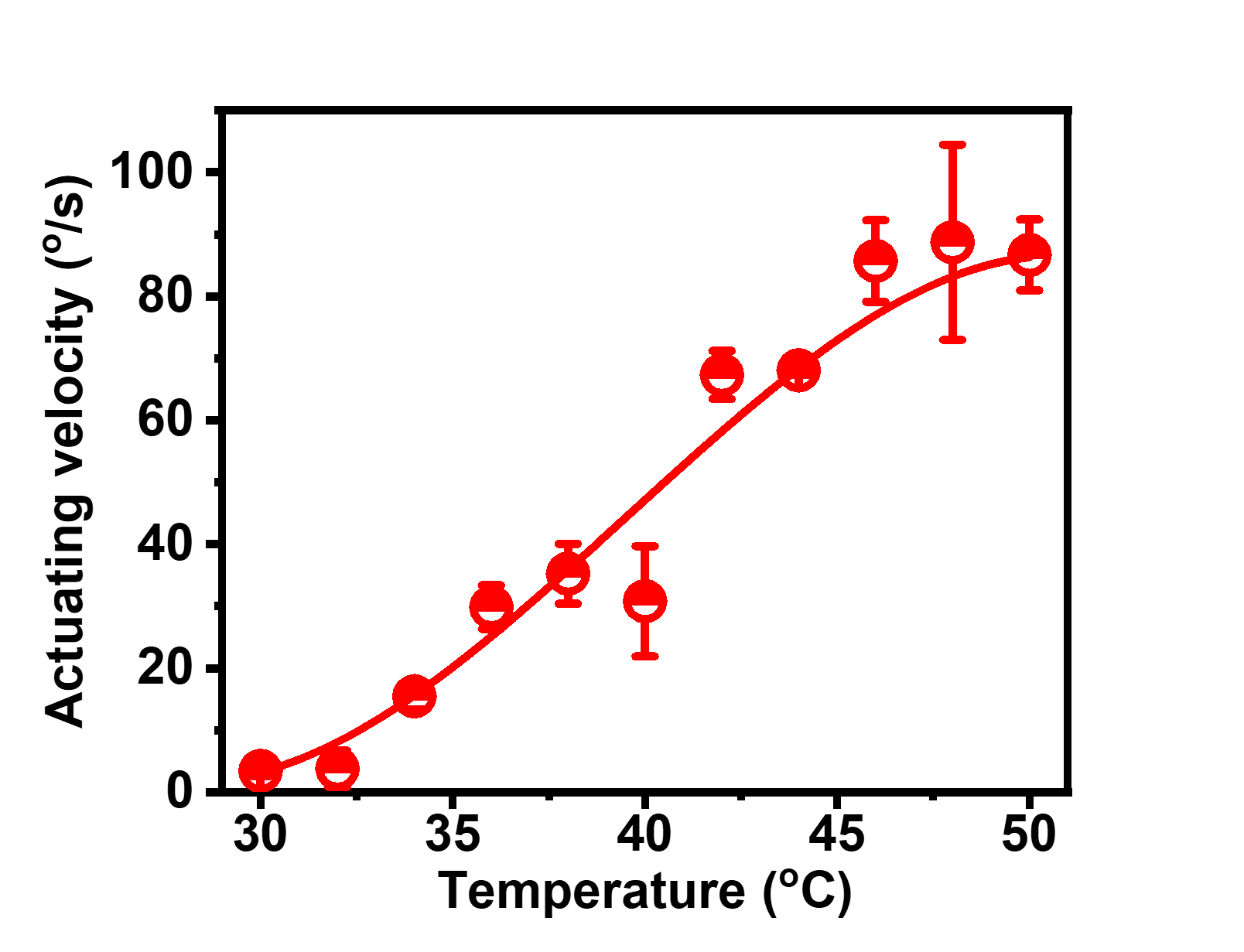


**Figure S7.** The actuating velocity of PNIPAm sponge based hydrogel actuator in different temperature.


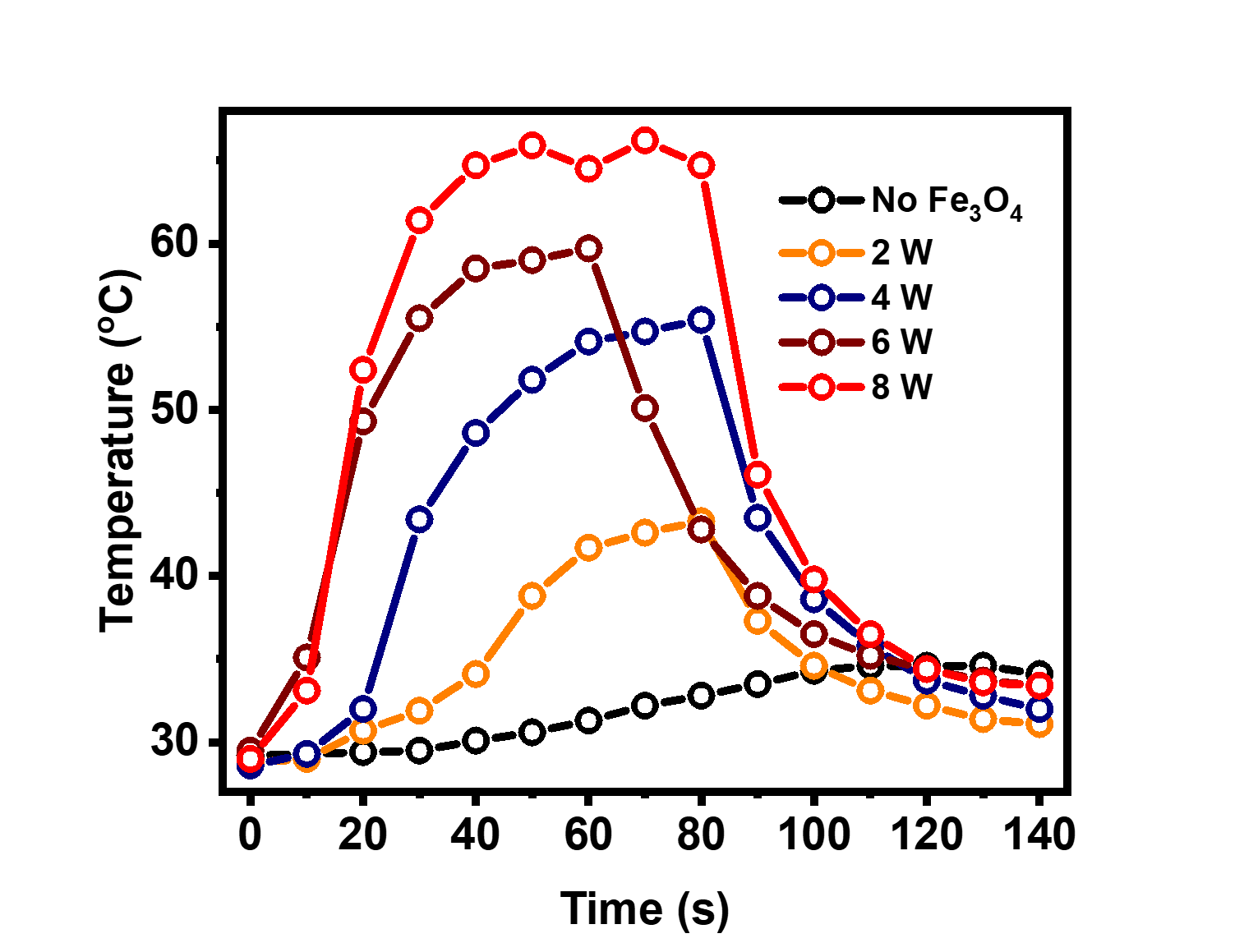


**Figure S8.** The photothermal curve of Fe_3_O_4_ NPs containing hydrogel when exposed under NIR light with different power.


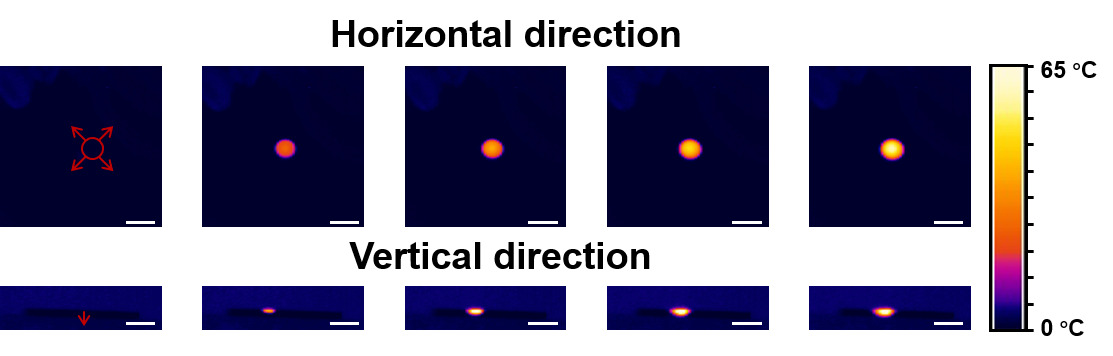


**Figure S9.** The heat flow condition of the hydrogel when a 6 W NIR irradiated on the surface of hydrogel. Scale bars: 1cm.


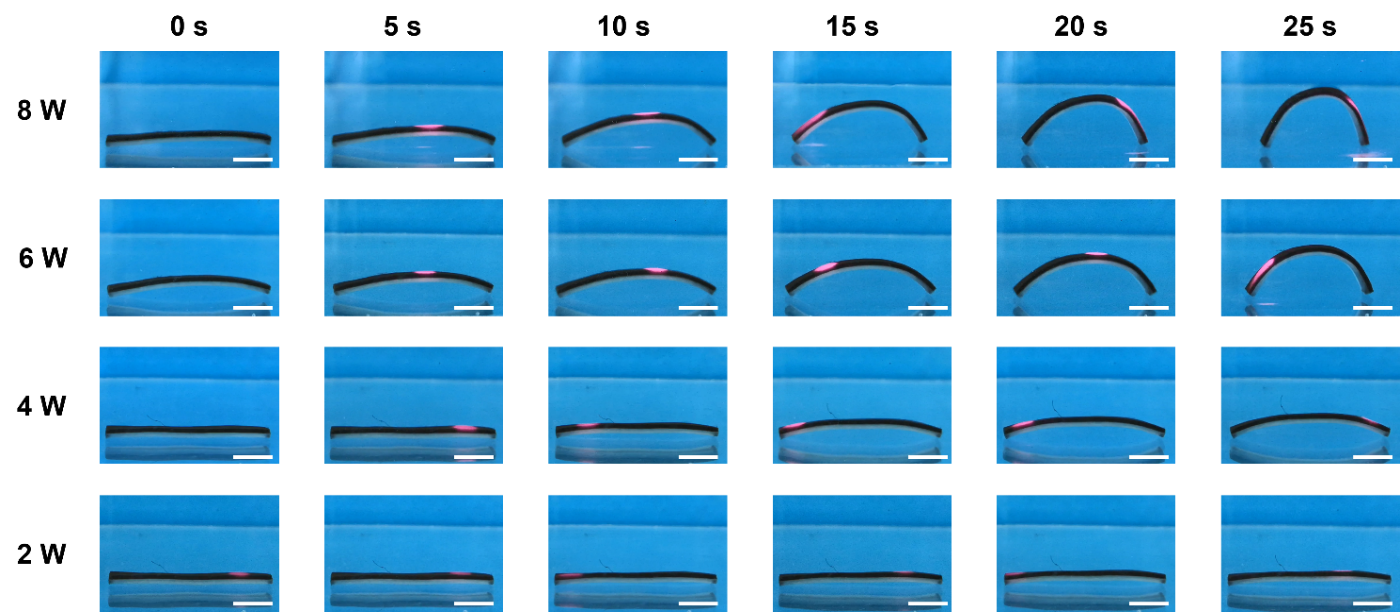


**Figure S10.** Images shown the photothermal responsive deformation process when exposed under NIR light with different power. Scale bars: 1 cm.


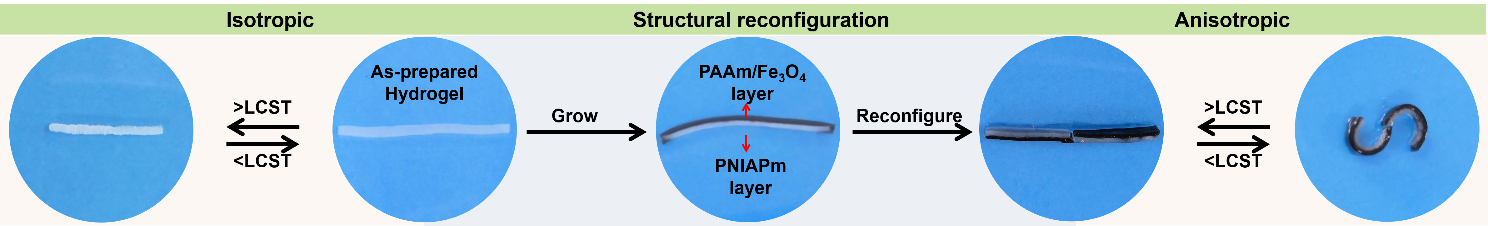


**Figure S11.** Images shown the structural reconfiguration of hydrogel from isotropic to anisotropic.


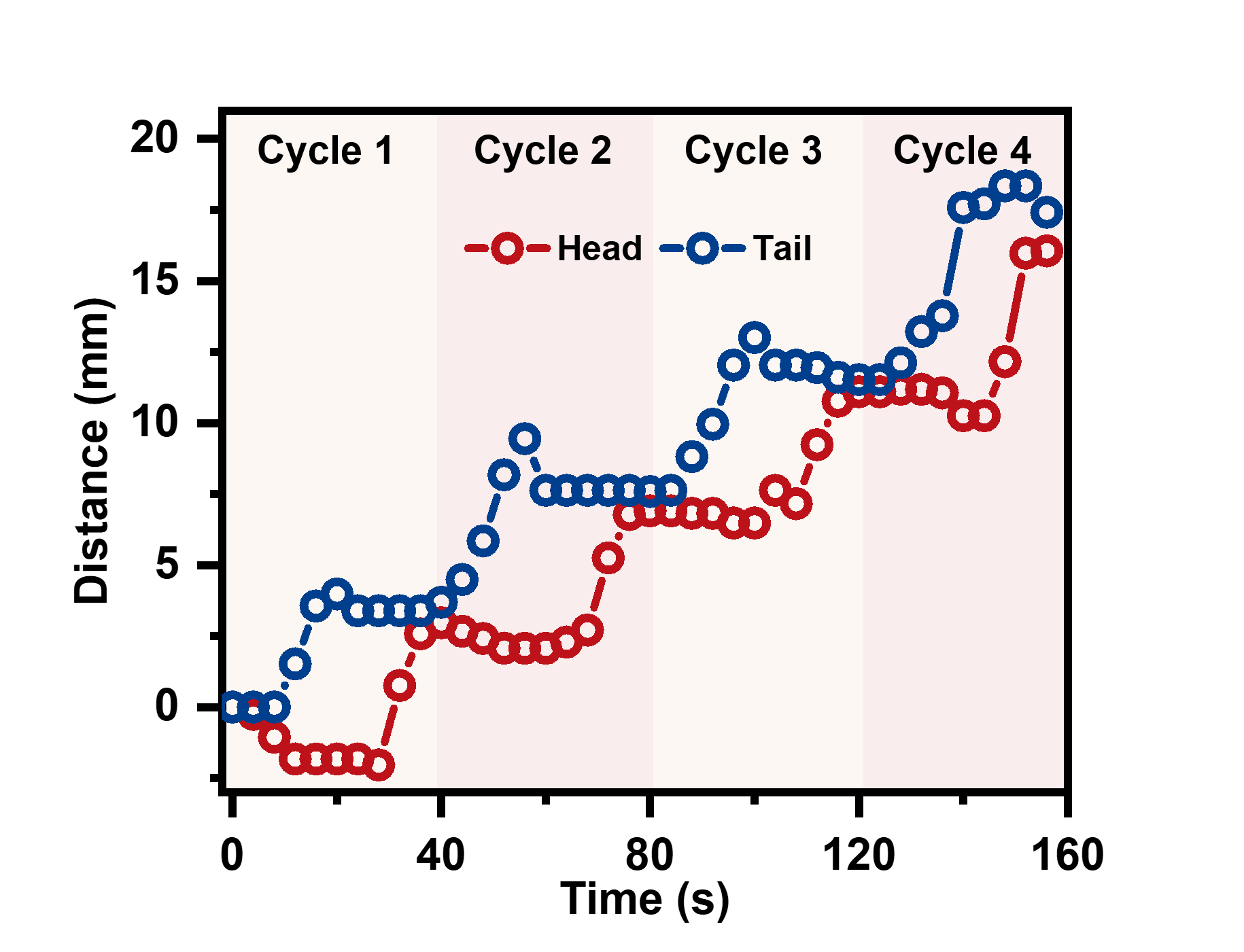


**Figure S12.** The feature trajectories of crawling cycles.


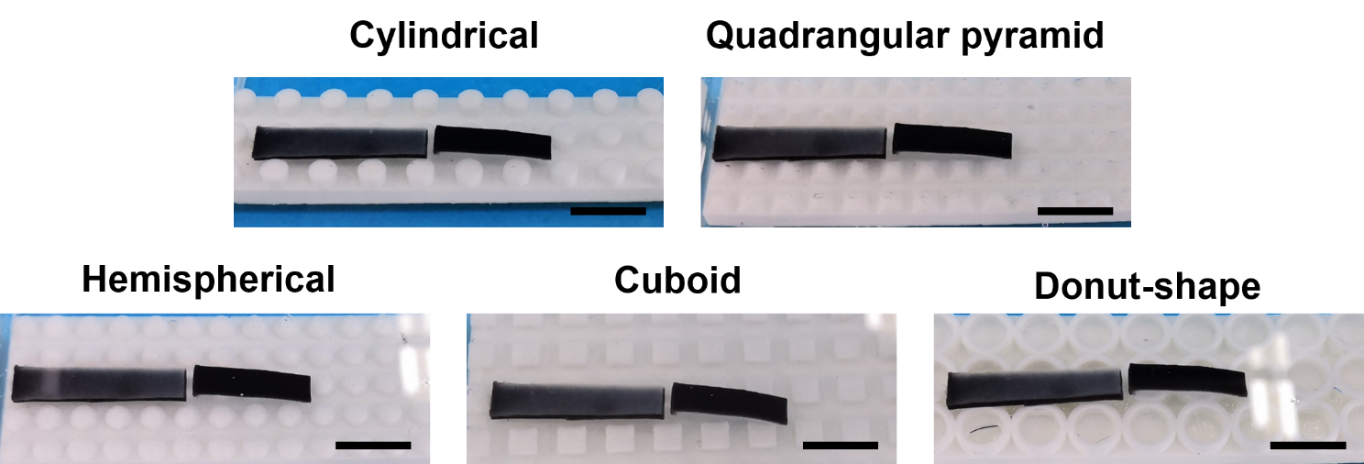


**Figure S13.** Images shown the different landform. Scale bars: 1 cm.


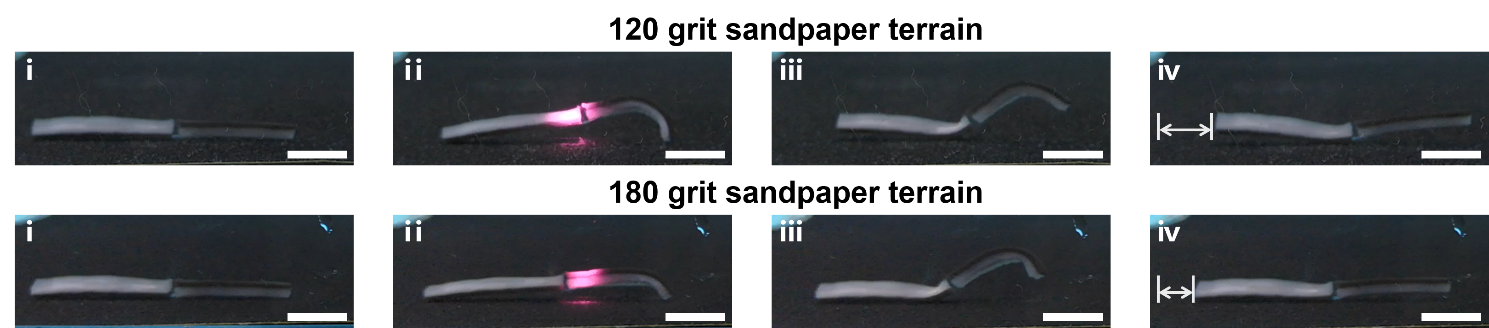


**Figure S14.** The locomotion process of hydrogel actuator above different sandpaper terrains. Scale bars: 1 cm.


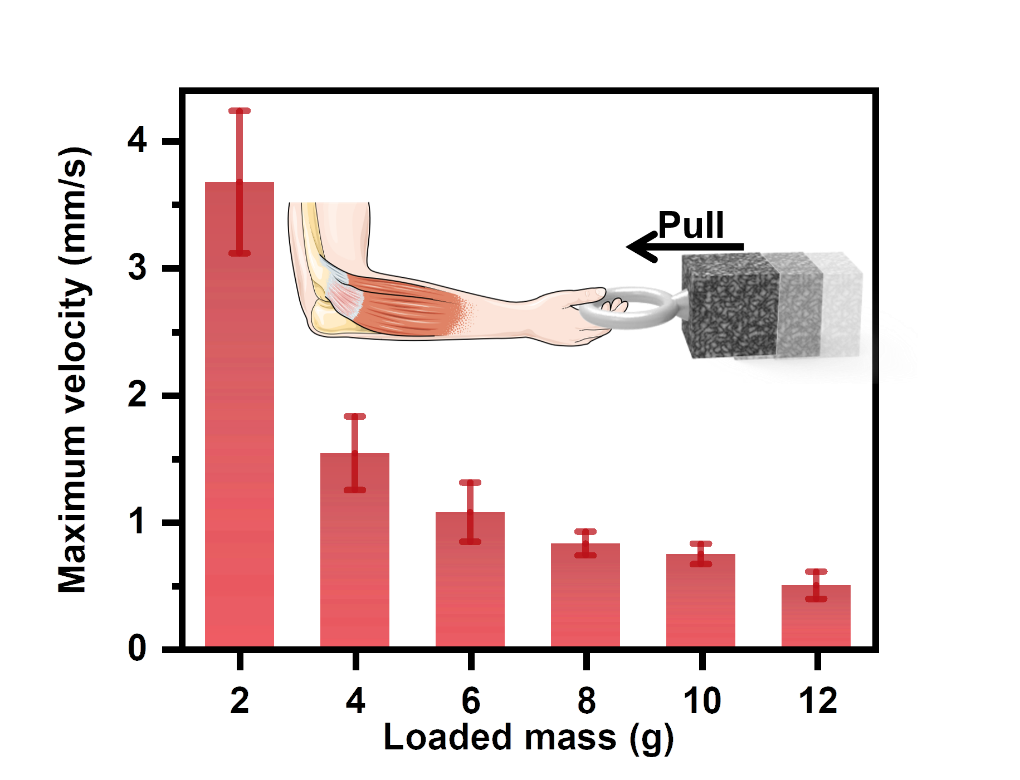


**Figure S15.** The energy export capability of hydrogel motor. The bilayer hydrogel actuator was utilized as hydrogel motor to pull the object with different weight.


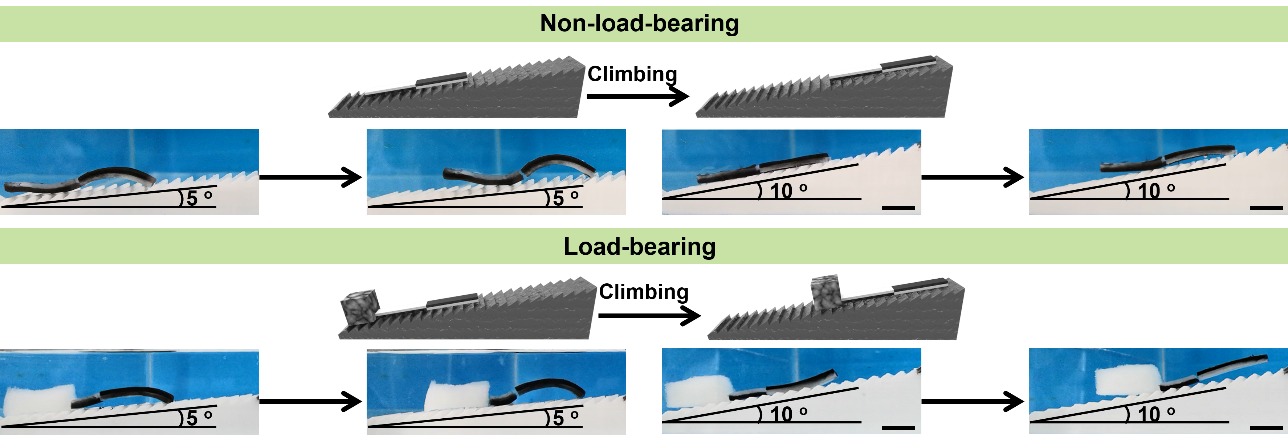


**Figure S16.** The hydrogel motor crawled on a slope loading or non-loading the object.


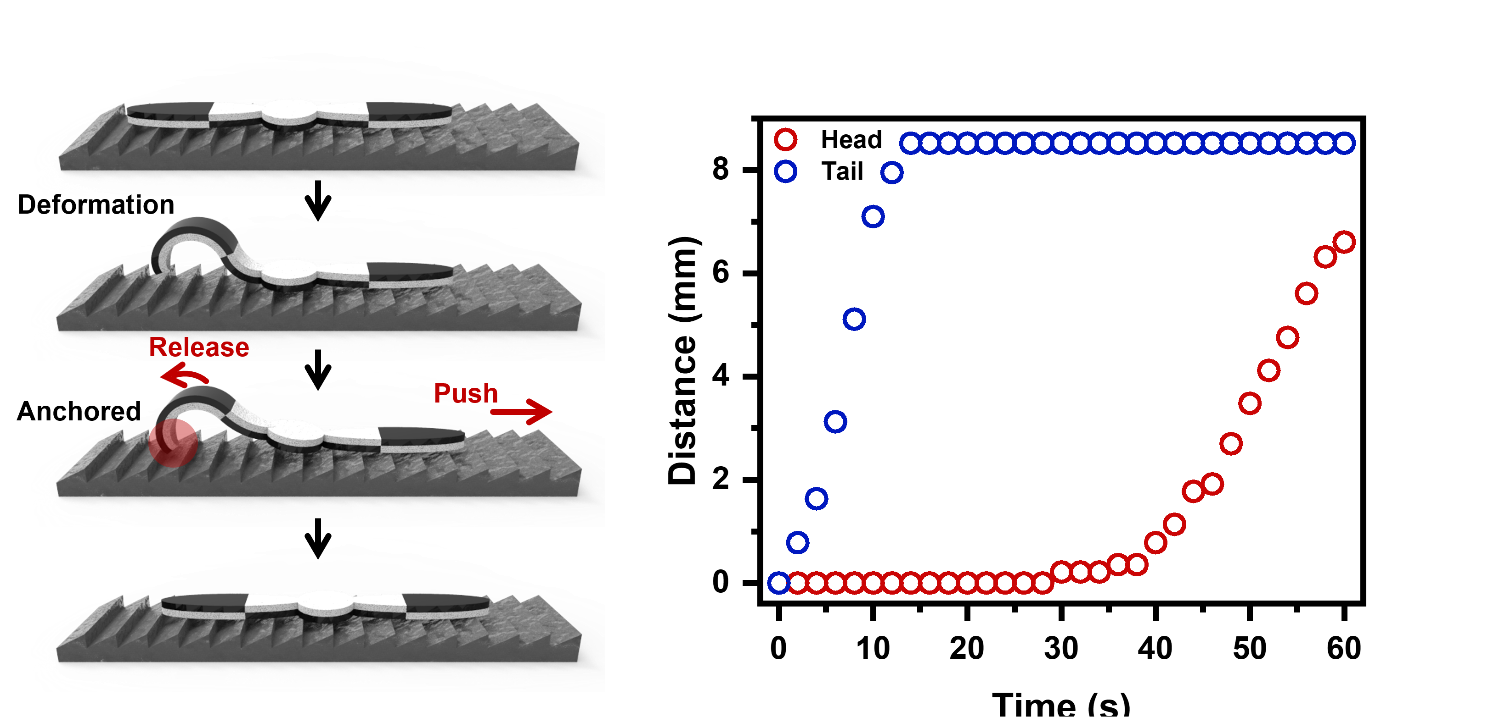


**Figure S17.** The feature trajectories of the head and tail of the hydrogel actuator in rear drive mode.


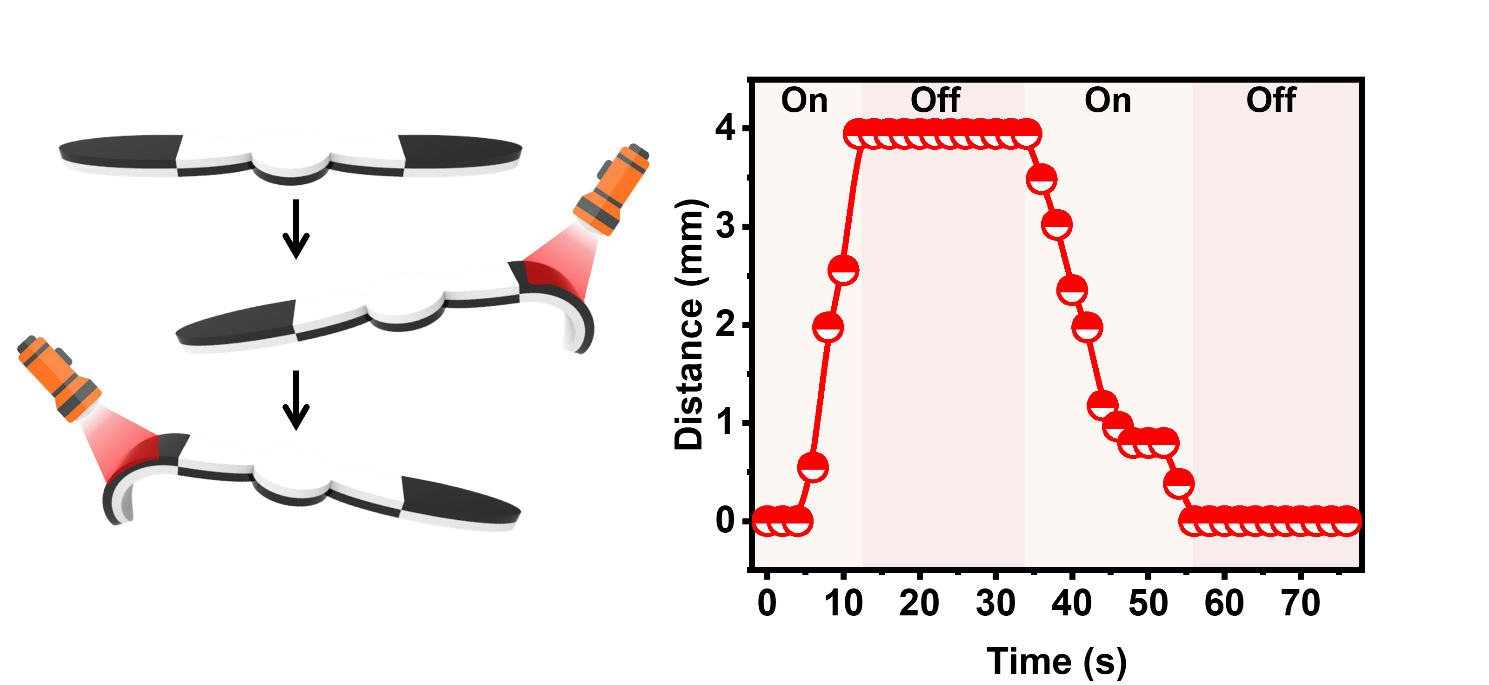


**Figure S18.** The two-way crawling of the bilayer hydrogel actuator.


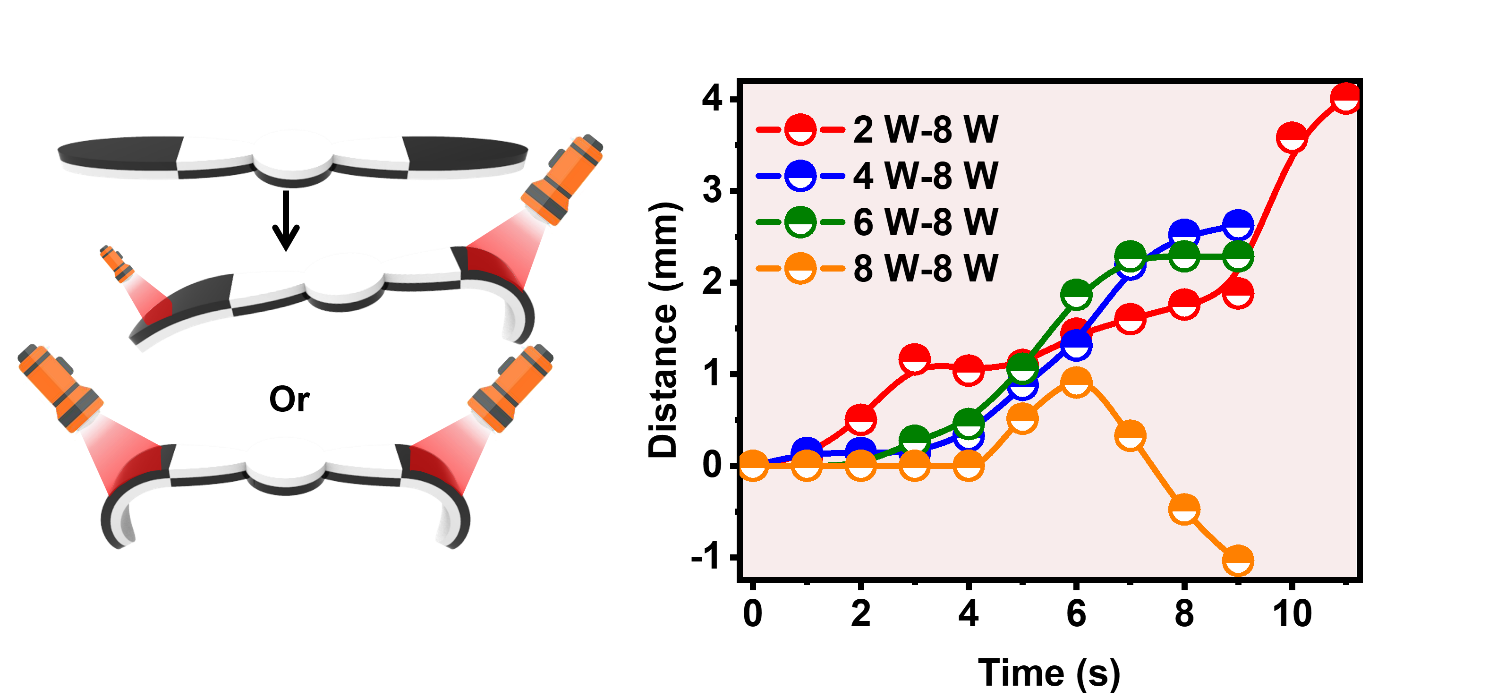


**Figure S19.** The competitive crawling of bilayer hydrogel actuator where a weak NIR and a strong NIR simultaneously irradiated on the left and right.


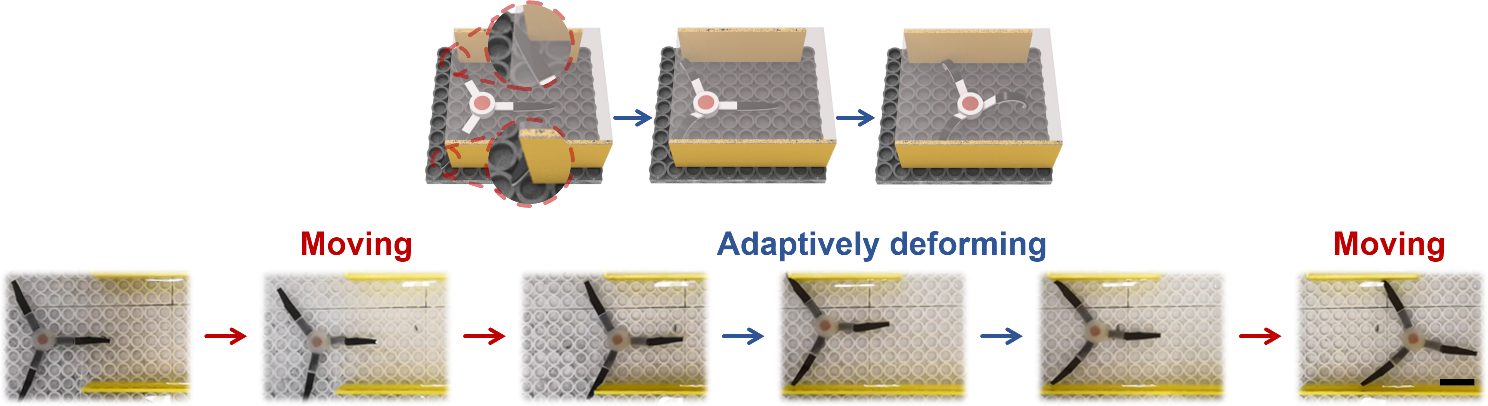


**Figure S20.** The composite soft robot could crawl and pass through narrow passage via adaptive deformation. Scale bar: 2 cm.


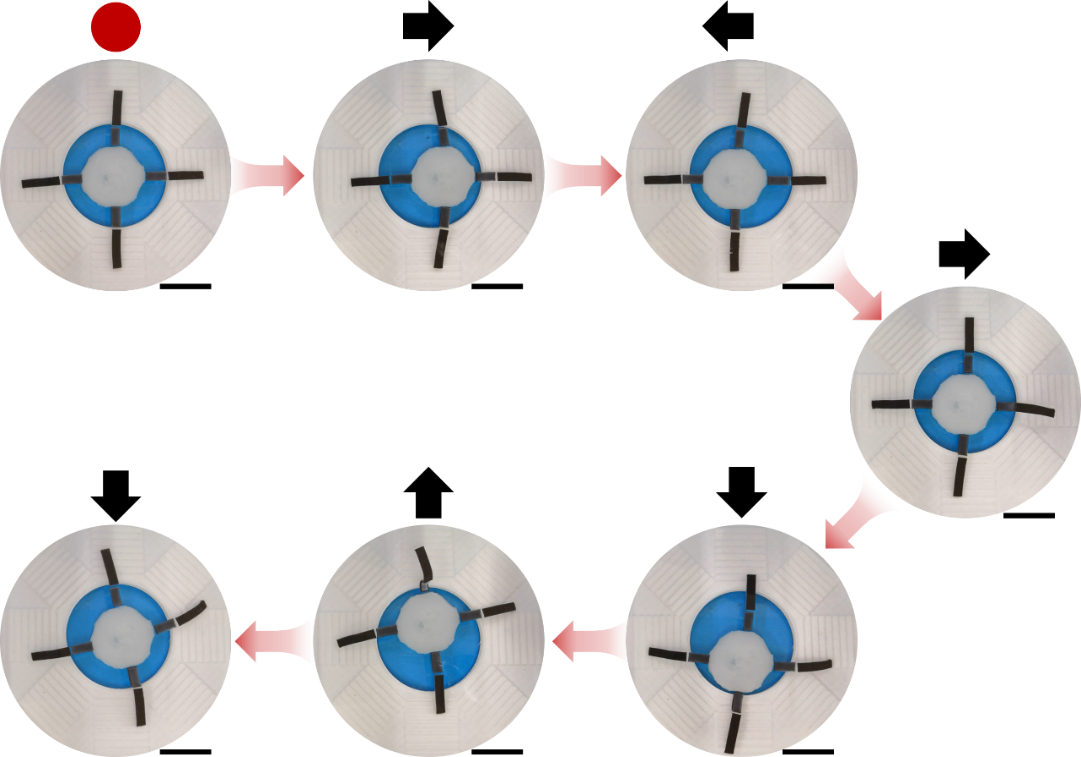


**Figure S21.** The moving process of PNIPAm sponge loaded with four hydrogel motors. Scale bars: 2 cm.


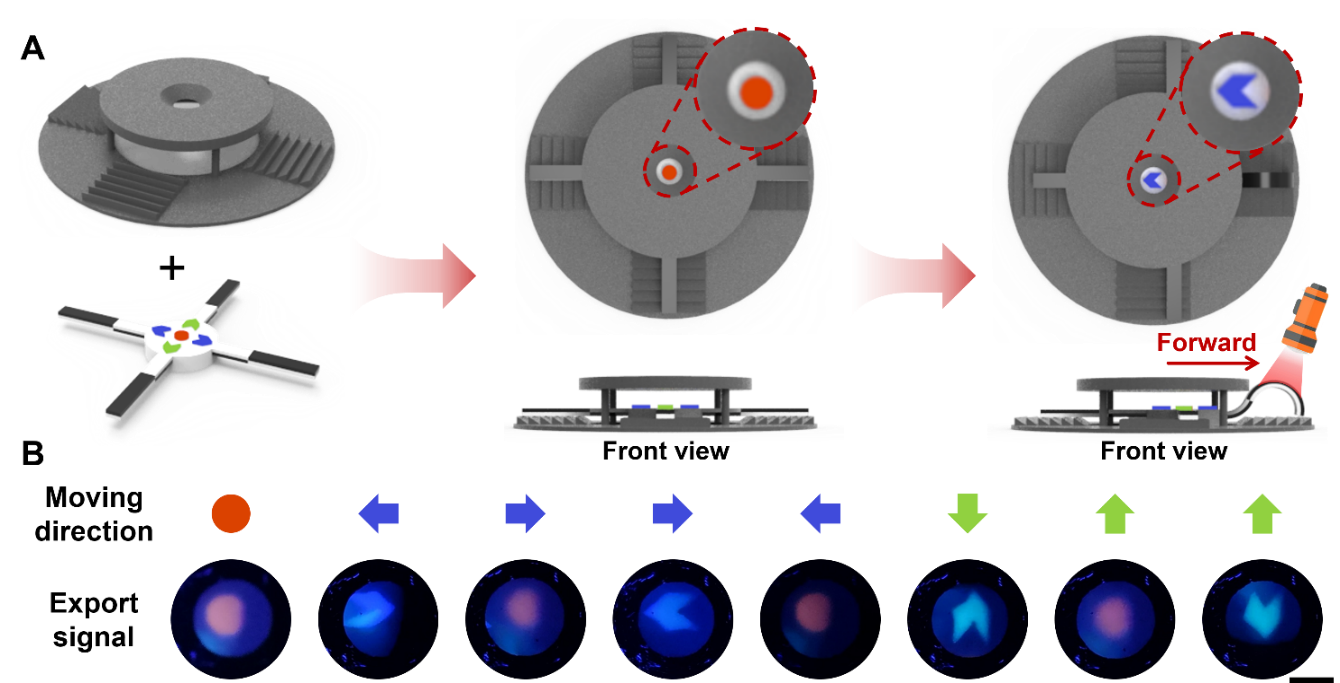


**Figure S22.** A) Multicolor fluorescent hydrogel grew on the surface of the PNIPAm sponge and assembled with four bilayer hydrogel motors via IDP to form a mechanical discoloration device. B) The mechanical discoloration device could transfer the fluorescent single by the mechanical movement of four hydrogel motors. Scale bar: 0.5 cm.


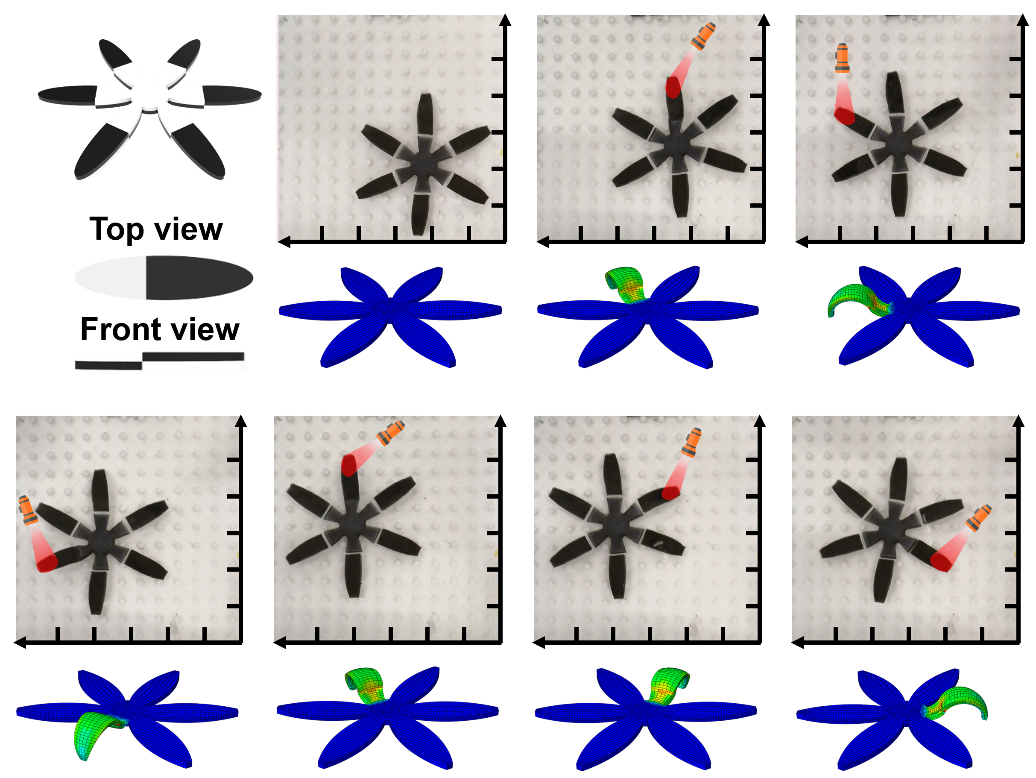


**Figure S23.** The freely locomotion of bilayer hydrogel actuator above 2D substrate. Scale bars: 5 mm.


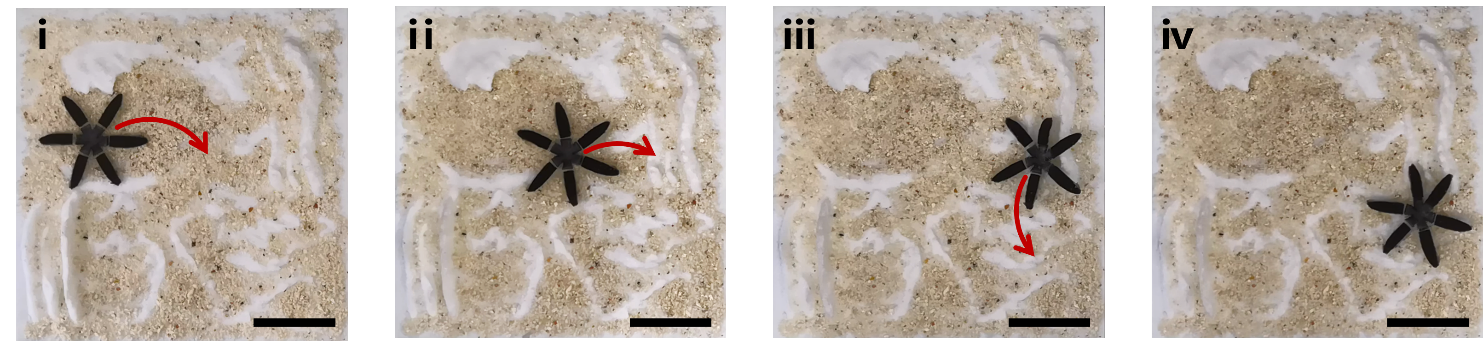


**Figure S24.** The freely locomotion of bilayer hydrogel actuator above 2D sandy terrain. Scale bars: 4 cm.

Supplementary Movie 1, Movie 2, Movie 3, Movie 4, Movie 5, Movie 6, Movie 7, Movie 8.

Supplementary Movie 1 shows the ultrafast thermo-responsiveness of PNIPAm sponge.

Supplementary Movie 2 shows the inchworm-inspired locomotion of hydrogel actuator.

Supplementary Movie 3 shows the 1D locomotion of hydrogel actuator above different terrains.

Supplementary Movie 4 Multi-mode of locomotion.

Supplementary Movie 5 shows the 2D locomotion of hydrogel actuator.

Supplementary Movie 6 shows the hydrogel motor moved the static object pass through the maze.

Supplementary Movie 7 shows the 2D freely crawl of hydrogel actuator.

Supplementary Movie 8 shows the 2D off-road locomotion of hydrogel actuator.
